# T-cell receptor/CD28-targeted immunotherapeutics selectively drive naive T-cell expansion to generate functional HIV-specific responses

**DOI:** 10.1128/jvi.00188-25

**Published:** 2025-08-05

**Authors:** April L. Mueller, Sara Lamcaj, Scott Garforth, Christopher Hiner, Darien Woodley, Kitt Paraiso, Tian Mi, Simon Low, Ben Youngblood, Steven C. Almo, Harris Goldstein

**Affiliations:** 1 Department of Microbiology and Immunology, Albert Einstein College of Medicine 2006, Bronx, New York, USA; 2 Department of Biochemistry, Albert Einstein College of Medicine 2006, Bronx, New York, USA; 3 Cellecta, Inc. 479172 , Mountain View, California, USA; 4 Department of Immunology, St. Jude Children's Research Hospital 5417, Memphis, Tennessee, USA; 5 Cue Biopharma 714617 , Boston, Massachusetts, USA; 6 Department of Pediatrics, Albert Einstein College of Medicine 2006, Bronx, New York, USA; St. Jude Children's Research Hospital, Memphis, Tennessee, USA

**Keywords:** HIV, immunology, T cells

## Abstract

Adoptive cell transfer (ACT), a promising immunotherapeutic approach, treats viral infections or cancer by *ex vivo* expansion and infusion of antigen-specific CD8+ T cells, respectively. However, its wider use is limited by logistical challenges associated with the conventional method of using patient-derived dendritic cells (DCs) loaded with peptides for *ex vivo* antigen-specific CD8+ T cell expansion. To overcome these limitations, we developed Immuno-STAT (IST), a dimeric protein scaffold that delivers peptide-specific T cell receptor (TCR) activation with or without CD28 costimulatory signals to expand CD8+ T cells specific for defined viral or cancer epitopes. In this proof-of-concept study, we demonstrate that anti-CD28-IST can selectively activate and expand polyfunctional cytotoxic CD8+ T cells from the naive repertoire, targeting the HIV-associated SL9 or melanoma-associated MART-1 epitopes. Naive MART-1-specific CD8+ T cells were reliably expanded by both peptide-loaded DCs and IST. In contrast, naive SL9-specific CD8+ T cells were expanded only by SL9-specific IST and not by conventional DC-based approaches, underscoring a unique ability of IST to stimulate some naive HIV-specific T cell responses. IST-derived SL9-specific CD8+ T cells exhibited potent cytotoxicity, diverse TCR clonotypes, and memory-differentiated phenotypes, marking a significant advance in generating antigen-specific T cells against HIV. The modular IST platform provides a scalable modality to stimulate naive CD8+ T cells to potentially mobilize preemptive CD8+ T cell responses against predicted immune escape variants, as well as subdominant conserved HIV epitopes to empower the development of innovative ACT, vaccine, and other immune strategies to advance treatments for HIV, other persistent viral infections, and cancer.

**IMPORTANCE:**

Adoptive transfer of *ex vivo*-expanded T cells with potent and broad anti-HIV activity may control HIV replication in people with HIV in the absence of antiretroviral therapy. To selectively activate and expand naive CD8+ cells targeting defined viral or cancer epitopes, we developed a unique protein architecture, termed Immuno-STAT, which delivers cognate peptide-specific T cell receptor (TCR) activation alone or in combination with CD28 costimulation. We demonstrated that polyfunctional cytotoxic CD8+ T cells specific for the HIV-associated SL9 or melanoma-associated MART-1 epitopes were expanded by αCD28-Immuno-STAT delivering peptide-specific TCR and CD28 signals, but not peptide-specific TCR signals alone. αCD28-Immuno-STAT-generated SL9-specific CD8+ T cells exhibited diverse TCR clonotypes, polyfunctionality, and potent SL9-specific cytotoxicity. Adoptive transfer of αCD28-Immuno-STAT-generated CD8+ T cells specific for defined HIV epitopes may provide the broad yet targeted responses specific for conserved HIV epitopes and predicted immune escape variants required to control HIV replication and provide a functional HIV cure.

## INTRODUCTION

New immunotherapeutic strategies are being developed and evaluated to generate clinically effective T cell responses to treat infectious diseases and cancer. One strategy, adoptive cell transfer (ACT), has been used to reconstitute anti-viral immunity in immunocompromised patients at high risk for developing life-threatening infections with viruses such as cytomegalovirus (CMV) (1) or Epstein-Barr virus (2) and is being optimized to treat cancer (3–5). ACT amplifies the patient’s immune response against virally infected or malignant cells by infusing autologous virus-specific or cancer T cells after *ex vivo* expansion by autologous antigen-presenting cells (APCs), such as mature dendritic cells (mDCs), to provide the large numbers of antigen-specific T cells required for functional activity after adoptive transfer (6). APCs possess a robust ability to present antigens and activate costimulatory receptors, delivering both the T cell receptor (TCR) and costimulatory signals necessary to effectively prime and significantly expand naive CD8+ T cells (7). However, there are several challenges that impede the broader use of mDCs as APCs to expand naive antigen-specific T cells for ACT, including logistical complexity, functional variability of generated DC, prohibitive cost for broad application, and variability in cell quality. Inhibitory ligands like TIM-3, HVEM, ILT4, and PD-L1 are also expressed by mDCs, which can inhibit T cell expansion and activity (8). Furthermore, large quantities of mDCs need to be generated before they can expand the tumor- or virus-specific T cells from naive precursors, thereby increasing the variability and costs and delaying the initiation of ACT. Therefore, mDCs do not provide an accessible and readily available “off-the-shelf” option for expanded clinical use or large-scale clinical trials.

Naive T cells have not yet encountered their cognate antigen, but once properly primed, they have the potential to respond robustly to their cognate antigen expressed by tumors or during viral infections. Naive CD8+ T cells are primed through three coordinated signals provided by antigen-presenting cells: (i) an antigen-specific activation signal selectively delivered through recognition of a peptide/MHC complex by its cognate TCR; (ii) a costimulatory signal elicited by engagement between APC cell surface ligands and coreceptor molecules expressed on T cells such as CD28, 4-1BB, or OX-40; and (iii) a proliferation and differentiation signal delivered by the binding of cytokines such as IL-15, IL-7, and IL-12 to their cognate receptors (9–11). The costimulatory signal is considered essential for the priming of naive T cells, as multiple studies have reported that TCR signaling in a naive T cell without a costimulatory signal induces clonal anergy, rendering the T cell unresponsive to subsequent activation (10, 12–14). Various strategies have aimed to deliver these two essential signals using artificial antigen-presenting cells (aAPCs), including nanoparticles (15–19), lipid scaffolds (20), HLA-Ig-coated beads (21, 22), biodegradable polymers (23), fibroblast-based artificial antigen-presenting cells (24, 25), genetically engineered APCs expressing costimulatory molecules (26), and engineered red blood cells (27).

We previously described a highly modular infusible immunomodulatory biologics platform, designated Immuno-STAT for Selective Targeting and Alteration of T cells (also referred to as synTac), designed to recapitulate delivery of TCR and costimulatory signals by antigen-presenting cells, which selectively expands and activates polyfunctional disease-specific cytotoxic CD8+ T cells (28–30). The IST framework provides stable antigen-specific engagement of TCR by covalent tethering of disease-specific peptide antigens to a single-chain linkage of β2 microglobulin to an MHC class I alpha chain (single-chain peptide-MHC, sc-pMHC), and the additional capacity to also deliver a costimulatory signal or cytokine signal by linkage to a ligand capable of binding to a co-stimulatory molecule (e.g., CD28 and 4-1BB) or cytokine receptor, respectively. The entire scaffold is constructed as an Fc-fusion protein to provide bivalent binding to the TCRs and costimulatory molecules with increased avidity and structural stability to facilitate manufacturability (29). ISTs bearing affinity-attenuated IL-2 were initially demonstrated to elicit expansion of naive and antigen-experienced HPV E7-specific CD8+ T cells in naive and immunized HLA-A2 transgenic mice, respectively (28, 29). Treatment of human peripheral blood mononuclear cells (PBMCs) with IL-2-linked ISTs tethered to distinct antigenic peptides stimulated the *in vitro* expansion of HPV E7-, MART-, and CMV-specific CD8+ T cells in the absence of additional costimulatory signals (28, 29). We previously reported that treatment with ISTs linked to an agonist CD28 scFv or single-chain trimeric 4-1BBL potently activated and expanded pre-existing CMV- and HIV-specific memory cytotoxic T lymphocytes *in vitro* in the presence of exogenous IL-2 and *in vivo* in NSG mice engrafted with human PBMCs (30). These examples underscore the modularity of the IST platform, where substituting the antigenic peptide allows targeting of antigen-specific T cells relevant to different disease indications, while incorporating distinct modulatory domains or cytokines supports differentiation of T cells with different functional activities. The clinical application of ISTs as potential immunotherapeutics is highlighted by the current evaluation of IL-2-linked ISTs presenting different antigenic peptides in clinical trials for recurrent metastatic head and neck cancer (NCT03978689) and WT-1-positive solid tumors (NCT05360680).

This study builds on previous findings by demonstrating the ability of Immuno-STAT constructs, engineered to incorporate sc-pMHC and CD28 costimulatory modules, to activate and expand human naive CD8+ T cells specific for the melanoma-associated MART-1 antigen, and most importantly, the HIV Gag-derived SL9 antigen in individuals without HIV. CD8+ T cells expanded using IST displayed robust polyfunctionality, including cytokine production, antigen-specific cytotoxicity against cancer or viral antigen-expressing cells, diverse TCR clonotypes, and an epigenetic profile consistent with memory T cell differentiation. While IST and mDC approaches successfully expanded MART-1-specific CD8+ T cells from the naive repertoire, naive SL9-specific CD8+ T cell activation expansion was achieved using SL9-specific IST constructs but not by treatment with DCs loaded with SL9 peptide. These findings highlight the distinct capability of IST to overcome barriers in expanding naive antigen-specific CD8+ T cells, particularly for challenging targets like SL9, a viral antigen from HIV. By enabling the generation of robust, functional SL9-specific T cells *ex vivo*, this proof-of-concept study establishes IST as a transformative platform for advancing immunotherapies targeting HIV and other infectious diseases.

## RESULTS

### IST constructs and experimental design

**Fig 1:**
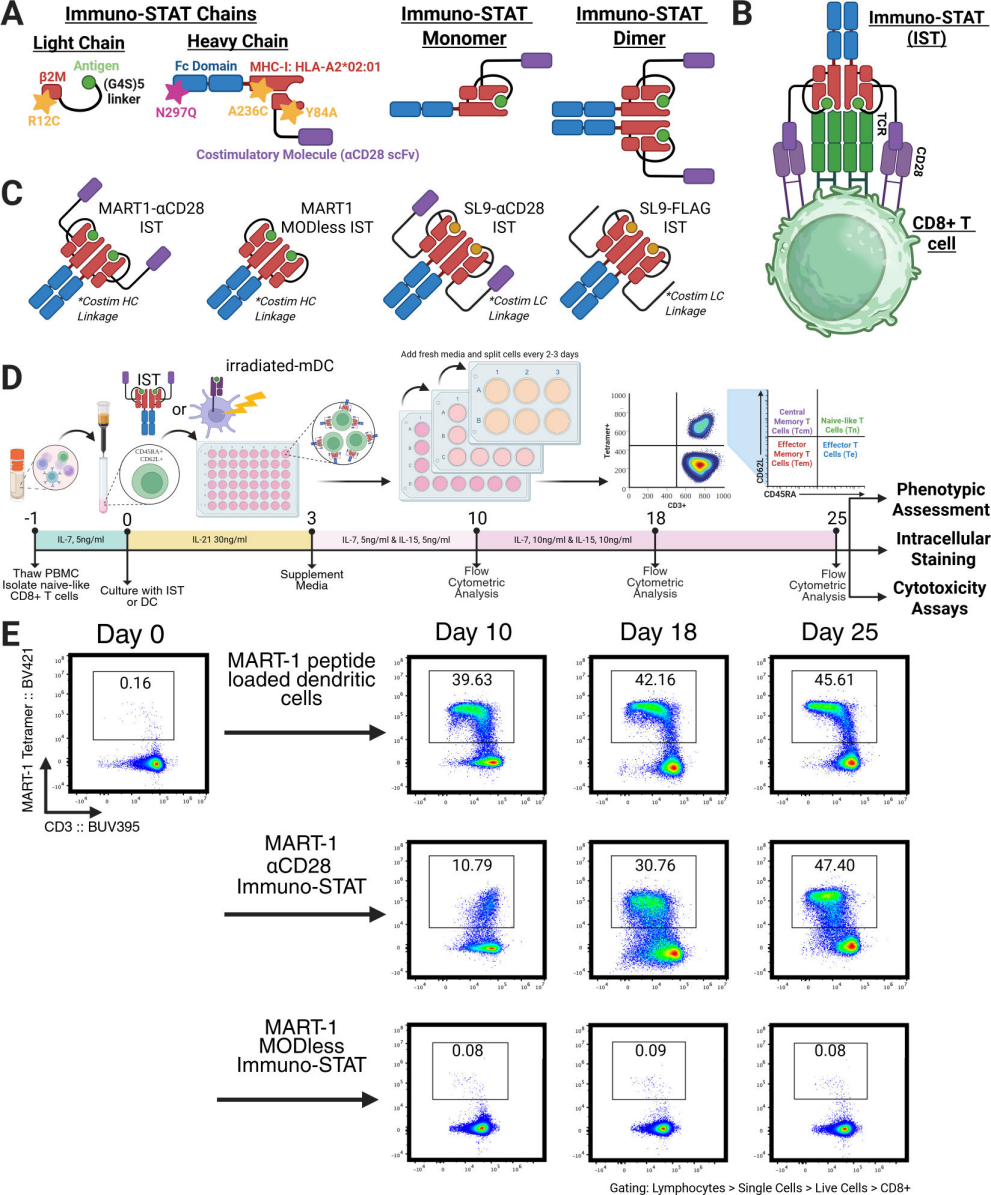
Structural representation of Immuno-STAT protein and experimental design. (**A**) Generalized IST Representations. (**B**) Graphic diagram of how IST mimics the immune synapse. (**C**) Designs of ISTs used. MART-1-ISTs contain either αCD28 or MODless variants in the HC-linked form, while SL9 ISTs contain either αCD28 or FLAG in the LC-linked form. (**D**) Schematic protocol for the expansion of naive CD8+ T cells using IST or mDC, detailed in Materials and Methods. (**E**) Representative flow plots of naive CD8+ T cells after treatment with αCD28 or MODless IST (100 nM) or peptide-loaded (2.5 µM) mature dendritic cells at the given time points.

The IST scaffold is a symmetric homodimeric Fc fusion protein composed of a light chain (LC) and heavy chain (HC) (Fig. 1A). The LC encodes a peptide antigen tethered to human β2M via a G3AS(G4S)2 linker and includes an R12C mutation to form a disulfide bond with the HC. The HC encodes an HLA-A*0201 MHC class I α chain with an A236C mutation for disulfide bonding and a Y84A mutation to accommodate the peptide linker, improving peptide presentation (31). The HC also includes an N297Q-mutated IgG1 Fc domain to prevent FcγR binding and antibody-dependent cellular cytotoxicity (32). This dimerized structure provides each IST molecule with the capacity to bind two TCRs and two costimulatory molecules, thereby triggering TCR and costimulatory signaling (Fig. 1B). SL9 ISTs, containing either αCD28 or a FLAG tag, were designed as previously described (30). For proof of concept, we also generated IST constructs presenting a modified MART-1 epitope (26-35, ELAGIGILTV) with increased binding affinity and stability (33), as MART-1-specific CD8+ T cells are abundant in the naive repertoire (~1 in 7,500 CD8+ T cells) and display a naive phenotype in healthy donors (34, 35). MART-1 ISTs were engineered with either an agonist αCD28 scFv (αCD28-MART-1-IST) or no costimulatory ligand (MODless-MART-1-IST) (Fig. 1C). We investigated whether ISTs could activate and expand primary human naive antigen-specific CD8+ T cells, particularly HIV-specific CD8+ T cells, and compared them to cells activated by mDCs using a validated protocol (36) (Fig. 1D). Highly purified naive CD8+ T cells were isolated using the Naive CD8+ T Cell Isolation Kit (Miltenyi Biotec, cat# 130-093-244), and antigen-specific expansion was monitored by tetramer staining and flow cytometry (Fig. 1E), with a representative gating strategy shown in Fig. S1A. The phenotype and functional activity of expanded MART-1-specific CD8+ T cells were evaluated on day 25.

### Delivery of a CD28 signal by αCD28 Immuno-STAT is required to activate and expand naive MART-1-reactive CD8+ cells

Highly purified naive CD8+ T cells (95%) were isolated by immunomagnetic sorting and displayed a naive phenotype (CD62L^+^, CD45RA^+^, and CD95⁻) with MART-1-specific precursors comprising ~0.15% of the population (Fig. S1B), consistent with previous reports in healthy HLA-A2+ donors (35, 37) (Fig. S1C). To determine the optimal IST concentration for activating and expanding MART-1-specific naive CD8+ T cells, we performed a dose-response analysis using αCD28-MART-1-IST or MODless-MART-1-IST (5–100 nM), measuring expansion by MART-1 tetramer staining on days 10, 18, and 25. Representative day 25 dot plots (Fig. S2A) and analyses of tetramer^+^ percentages (Fig. S2B), cell counts/mL (Fig. S2C), and fold change (Fig. S2D) revealed that αCD28-MART-1-IST, but not MODless-IST, expanded MART-1-specific cells in a dose-dependent manner, most potently at a concentration of ≥50 nM. On day 25, treatment with αCD28-MART-1-IST (50 nM) expanded MART-1-specific cells to 33% of total CD8+ cells, corresponding to ~500,000 tetramer^+^ cells/mL, a 500-fold increase from day 0.

Next, we evaluated cytokine conditions for naive CD8+ T cell expansion with αCD28-MART-1-IST (100 nM), comparing IL-21/7/15 (IL-21 for 3 days, followed by IL-7 and IL-15), IL-2 (100 U/mL), or IL-15 (10 ng/µL) alone. Only the IL-21/7/15 protocol supported significant expansion at all time points tested (Fig. S2E) and was adopted for all subsequent experiments. These findings demonstrate that the αCD28-IST platform effectively expands and differentiates naive CD8+ T cells when combined with the IL-21/7/15 cytokine regimen.

### Comparison of the expansion of MART-1-specific cells after IST or autologous mDC treatment

To compare αCD28-MART-1-IST-induced expansion of MART-1-specific naive CD8+ T cells to established methods, we utilized autologous mDCs pulsed with MART-1 (2.5 µM, MART-1-mDCs). mDCs were derived from donor monocytes as described (36), with reduced CD14 and increased CD83 expression confirming their phenotype (Fig. S3A). Naive CD8+ T cells were cultured with MART-1-mDCs (1:4 mDC:T ratio) or αCD28-MART-1-IST (100 nM) using identical protocols, including media supplementation and splitting (detailed in Materials and Methods).

**Fig 2:**
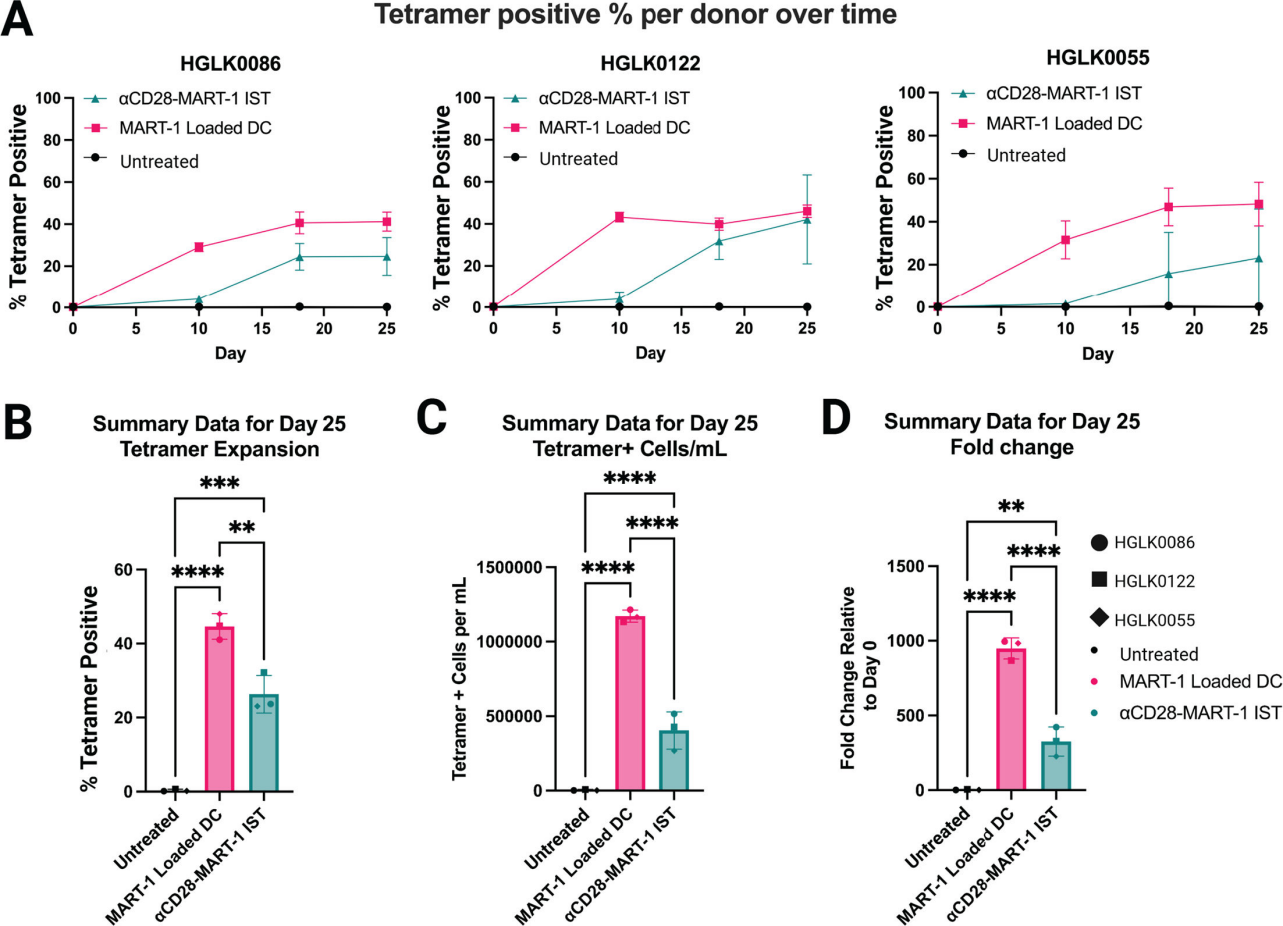
IST expands MART-1-specific CD8+ T cells from the naive repertoire in multiple donors. (**A**) Expansion plots over time for each donor. Line graphs depicting the percentage of tetramer positive at each time point tested (days 0, 10, 18, and 25) after treatment with 100 nM αCD28-MART-1-IST or MART-1-mDC. Each data point represents the mean ± SD of three to five biological replicates for each donor (*n* = 3). Each donor was tested in separate experiments on different days. (**B**) Summary data for the percentage of tetramer expansion on day 25. Bar plot representing summary results for tetramer expansion on day 25. Each data point represents an average of three to five biological replicates for each donor (*n* = 3). (**C**) Summary data for tetramer+ cell count per milliliter on day 25. Bar plot showing tetramer counts on day 25 of expansion, calculated as in panel B. Each data point represents an average of three to five biological replicates for each donor (*n* = 3). (**D**) Summary data for fold change on day 25. Bar plot representing summary results for fold change, calculated as in panel B. Each data point represents an average of three to five biological replicates for each donor (*n* = 3). Significance for panels **B–D** was determined by ordinary one-way ANOVA followed by Tukey’s multiple comparisons test. All statistical analysis was done in GraphPad Prism 10.4.0 (*P < 0.05, **P < 0.01, ***P < 0.001, and ****P < 0.0001).

Expansion kinetics of MART-1-specific CD8+ T cells from three donors were evaluated by tetramer staining at specified time points (Fig. 2A). After 25 days, MART-1-specific CD8+ T cells were significantly expanded with MART-1-mDCs (*P* < 0.0001) and αCD28-MART-1-IST (*P* < 0.001) compared to untreated cells, as measured by MART-1-specific CD8+ T cell percentage (Fig. 2B), cell count/mL (Fig. 2C), and fold change from day 0 (Fig. 2D). On day 25, ~ 26% of CD8+ T cells were MART-1-specific after αCD28-MART-1-IST treatment (24%, 32%, and 23% in donors HGLK0086, HGLK0122, and HGLK0055, respectively), compared to ~45% after MART-1-mDC treatment (41%, 45%, and 48%, respectively). These results demonstrate the ability of αCD28-MART-1-IST to activate and expand MART-1-specific CD8+ T cells across multiple donors, although greater expansion of MART-1-specific cells was observed after MART-1-mDC treatment compared to αCD28-MART-1-IST treatment in all donors evaluated.

### Comparison of phenotype and functional activity of MART-1-specific cells expanded by αCD28-MART-1-IST or MART-1-mDC

**Fig 3:**
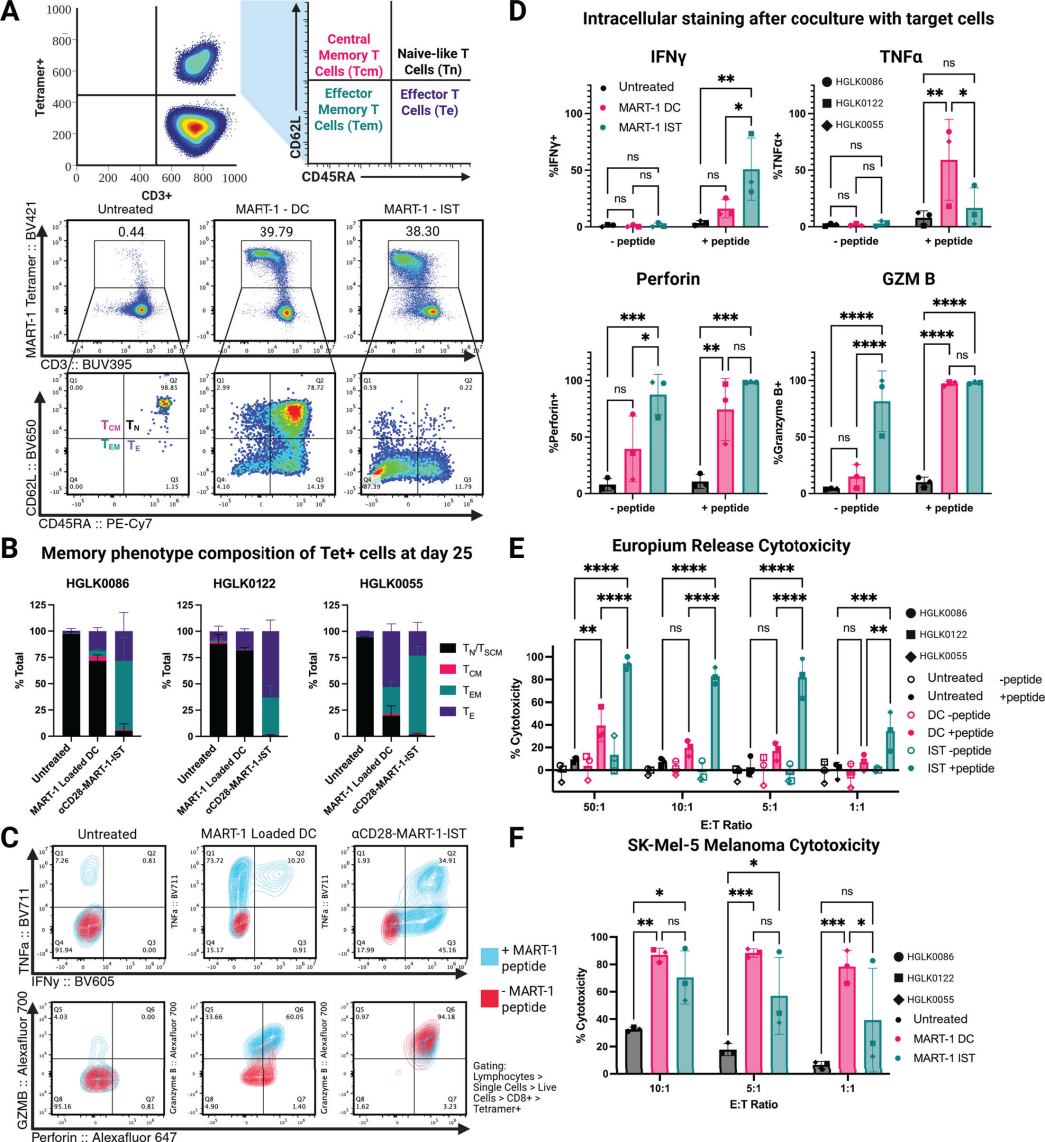
Treatment of naive CD8+ T cells by MART-1-IST yields differentiated cells that are polyfunctional and cytotoxic. (**A**) Representative flow cytometry plots on day 25 of culture of phenotype gating strategy. Tetramer-positive cells were stratified by their expression of CD62L and CD45RA as follows: Tnaive-like: CD62L+ CD45RA+ (black), Tcentral memory: CD62L+ CD45RA− (pink), Teffector memory: CD62L− CD45RA− (green), and Teffector: CD62L− CD45RA+ (purple). (**B**) Memory phenotype composition analysis for each donor on day 25. Stacked bar plot shows the memory composition of tetramer-positive cells on day 25 after stimulation with either peptide-loaded mDC or 100 nM antiCD28-MART-1-IST. Graphs were generated from three to five biological replicates for each donor, with each stack representing the mean ± SD. Experiments for each donor were conducted separately. (**C**) Representative flow plots for intracellular cytokine staining. Contour plots show tetramer-positive cells stimulated by either MART-1 peptide-loaded T2 cells (blue) or vehicle control (red). Contour plots assess dual expression of interferon-gamma and TNF-alpha (top) or perforin and granzyme B (bottom). (**D**) ICS summary data. Bar plots showing summary data for intracellular staining for cytokine expression. Each data point represents averaged results from three biological replicates from *n* = 3 donors. Experiments for each donor were conducted separately. (**E**) Europium release cytotoxicity assay. Summary results from europium-based release assay against peptide-loaded or vehicle control-loaded T2 cells by untreated, mDC-treated, or IST-treated cells. Each data point represents averaged results from three biological replicates from *n* = 3 donors. Experiments for each donor were conducted separately. (**F**) Melanoma cell line cytotoxicity summary data. Summary results from flow cytometry-based cytotoxicity assay against SK-Mel-5 melanoma cell line by untreated, mDC-treated, or IST-treated cells. Each data point represents averaged results from *n* = 3 biological replicates from *n* = 3 donors. Experiments for each donor were conducted separately. All statistical analysis was done in GraphPad Prism 10.4.0. Significance was estimated by a two-way ANOVA, and group differences at each condition were computed and assessed via analyses of simple effects, using the error term and degrees of freedom from the whole design (*P < 0.05, **P < 0.01, ***P < 0.001, and ****P < 0.0001).

We compared the phenotype and functionality of MART-1-specific CD8+ T cells expanded by αCD28-MART-1-IST and MART-1-mDC. On day 25, memory phenotypes were assessed using CD62L and CD45RA expression to classify cells into T cell subsets, central memory (TCM), effector memory (TEM), naive or stem cell memory (TN/SCM), and effector (TE) (38, 39) (Fig. 3A). MART-1-mDC-expanded cells were predominantly TN/SCM in donors HGLK0086 (71.8%) and HGLK0122 (82.1%) or TE in donor HGLK0055 (53.0%). In contrast, αCD28-MART-1-IST-expanded cells were predominantly TEM in donors HGLK0086 (66.0%) and HGLK0055 (74.0%) or TE in donor HGLK0122 (63.0%) (Fig. 3B). Untreated cells remained TN/SCM throughout the culture period. These results indicate that αCD28-MART-1-IST treatment induces a more differentiated phenotype than MART-1-mDC.

Functional activity was evaluated on day 25 by measuring cytokine (TNFα and IFNγ) and lytic protein (perforin and granzyme B) production after stimulation with MART-1 peptide-loaded T2 cells (Fig. 3C). αCD28-MART-1-IST-expanded cells produced significantly more IFNγ (*P* < 0.05), while MART-1-mDC-expanded cells produced more TNFα (*P* < 0.05) (Fig. 3D). Both treatments induced antigen-specific responses, with no significant cytokine production in controls. αCD28-MART-1-IST-expanded cells expressed significantly higher baseline perforin and granzyme B levels (*P* < 0.001 and *P* < 0.0001), reflecting their TEM phenotype. MART-1-mDC-expanded cells exhibited moderate perforin and low granzyme B at baseline, with increased expression upon stimulation.

Cytotoxicity was assessed using a 3-hour europium-release assay. αCD28-MART-1-IST-expanded cells exhibited significant killing of peptide-loaded T2 cells at all E:T ratios (50:1, 10:1, 5:1, and 1:1; *P* < 0.001 or *P* < 0.0001) compared to untreated controls, with minimal activity against unloaded T2 cells. MART-1-mDC-expanded cells showed significant cytotoxicity (*P* < 0.01) only at 50:1 (Fig. 3E). Against the SK-Mel-5 melanoma cell line, both treatments induced significant target cell killing at high E:T ratios (10:1 and 5:1), with no significant differences observed (Fig. 3F). While αCD28-MART-1-IST-expanded cells displayed more robust cytotoxicity in short-term assays, MART-1-mDC-expanded cells performed comparably in longer assays, possibly due to delayed effector function development. These findings highlight the capacity of αCD28-MART-1-IST to efficiently expand MART-1-specific CD8+ T cells with potent cytotoxic activity against naturally processed MART-1 antigen.

### αCD28-MART-1-IST expanded MART-1-specific cells are more differentiated than those expanded by MART-1-mDC treatment based on their epigenetic methylation profile

**Fig 4:**
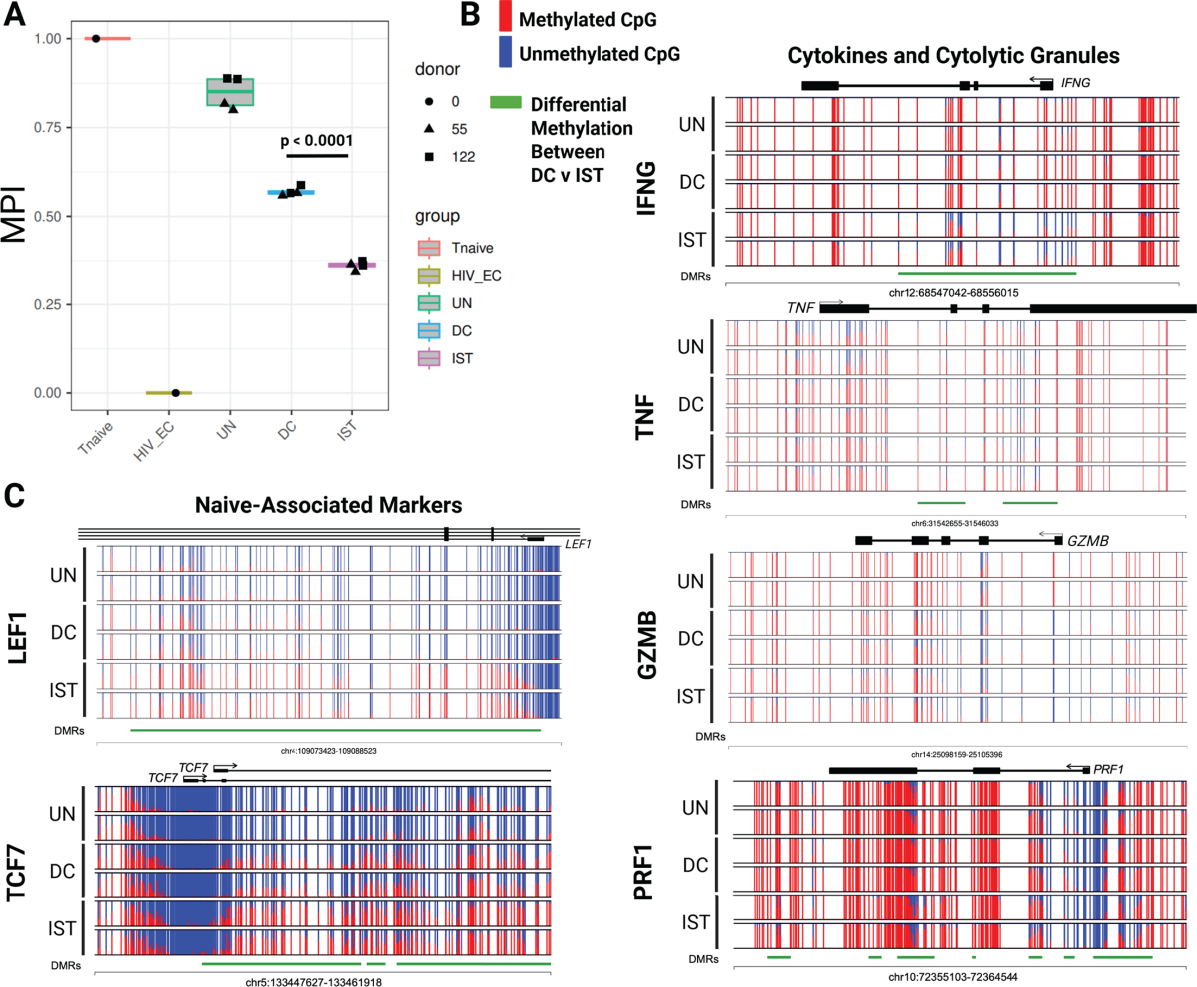
Epigenetic signatures of MART-1-IST treatment are consistent with differentiated cells. (**A**) T cell multipotency index. T cell multipotency score was generated from 0 to 1 for each indicated sample (UN, untreated; DC, dendritic cell treated; and IST, MART-1 Immuno-STAT treated) and compared to true naive (Tnaive, 1) and terminally exhausted (HIV_EC, 0) reference samples. Each dot represents one technical replicate from two donors shown as triangles or squares. (**B**) Normalized plots of CpG methylation from donor HGLK005 at sites surrounding and within differentially methylated regions of effector molecules (IFNG, TNF, GZMB, and PRF1) and (**C**) naive associated transcription factors (LEF1 and TCF7) obtained from EM Seq analysis. Red and blue lines depict methylated and unmethylated CpG sites, respectively, while green lines depict differentially methylated regions between IST and mDC. Significance was calculated by ordinary one-way ANOVA followed by Tukey’s multiple comparisons test using GraphPad Prism 10.4.0.

We used whole-genome methylation profiling (EM Seq) to compare the differentiation-associated programs of highly purified MART-1-specific CD8+ T cells expanded by αCD28-MART-1-IST or MART-1-mDC in two donors. Differentiation status was assessed using the multipotency index, which ranges from 0 (terminally differentiated HIV-specific CD8+ T cells) to 1 (freshly isolated naive CD8+ T cells), based on the methylation status of 245 CpG sites from standardized controls, as previously reported (40) (Fig. 4A). MART-1-specific CD8+ T cells expanded by αCD28-MART-1-IST exhibited a significantly lower multipotency index (~0.36) compared to MART-1-mDC-expanded cells (~0.57, *P* < 0.0001), indicating greater differentiation. Untreated samples had an index of ~0.81, likely reflecting low-level differentiation during 25-day IL21/7/15 cytokine culture (41, 42).

We next generated CpG plots for a select number of differentially expressed genes between DC- and IST-stimulated cells. These plots display the methylation status of individual CpG sites across key gene loci, with each row representing a single sample in duplicate, and each graph representing a specific CpG site. Unmethylated (open, blue) sites are typically associated with active gene expression, while methylated (closed, red) sites indicate transcriptional repression. The pattern and density of methylation across cells provide insights into the epigenetic programming of the CD8^+^ T cells, distinguishing effector, memory, and exhausted states. Pairwise comparison of differentially methylated regions (DMRs) revealed 6,199 regions with >20% methylation differences, including effector-associated loci such as IFNG, TNF, and PRF1 (Fig. 4B). The IFNG locus was significantly less methylated in αCD28-MART-1-IST-treated cells, aligning with higher IFNγ production observed in intracellular cytokine assays. Reduced methylation at IFNG and PRF1 suggests that αCD28-MART-1-IST-expanded cells are transcriptionally poised for robust effector responses, consistent with their polyfunctional phenotype. No significant DMRs were observed in GZM B. Stem cell-associated factors TCF7 and LEF1 showed higher methylation in αCD28-MART-1-IST-treated cells, reflecting their more differentiated phenotype. CpG methylation plots from donor HGLK0086 (Fig. 4) and comparable results from donor HGLK0122 (Fig. S4) demonstrate consistent epigenetic differences between αCD28-MART-1-IST- and MART-1-mDC-expanded cells. The increased demethylation of effector-associated loci and differentiation profiles of αCD28-MART-1-IST-expanded cells correlate with the phenotypic and functional analyses presented earlier.

### MART-1-specific CD8+ T cells expanded by αCD28-MART-1-IST display lower TCR diversity than those expanded by MART-1-mDC

**Fig 5:**
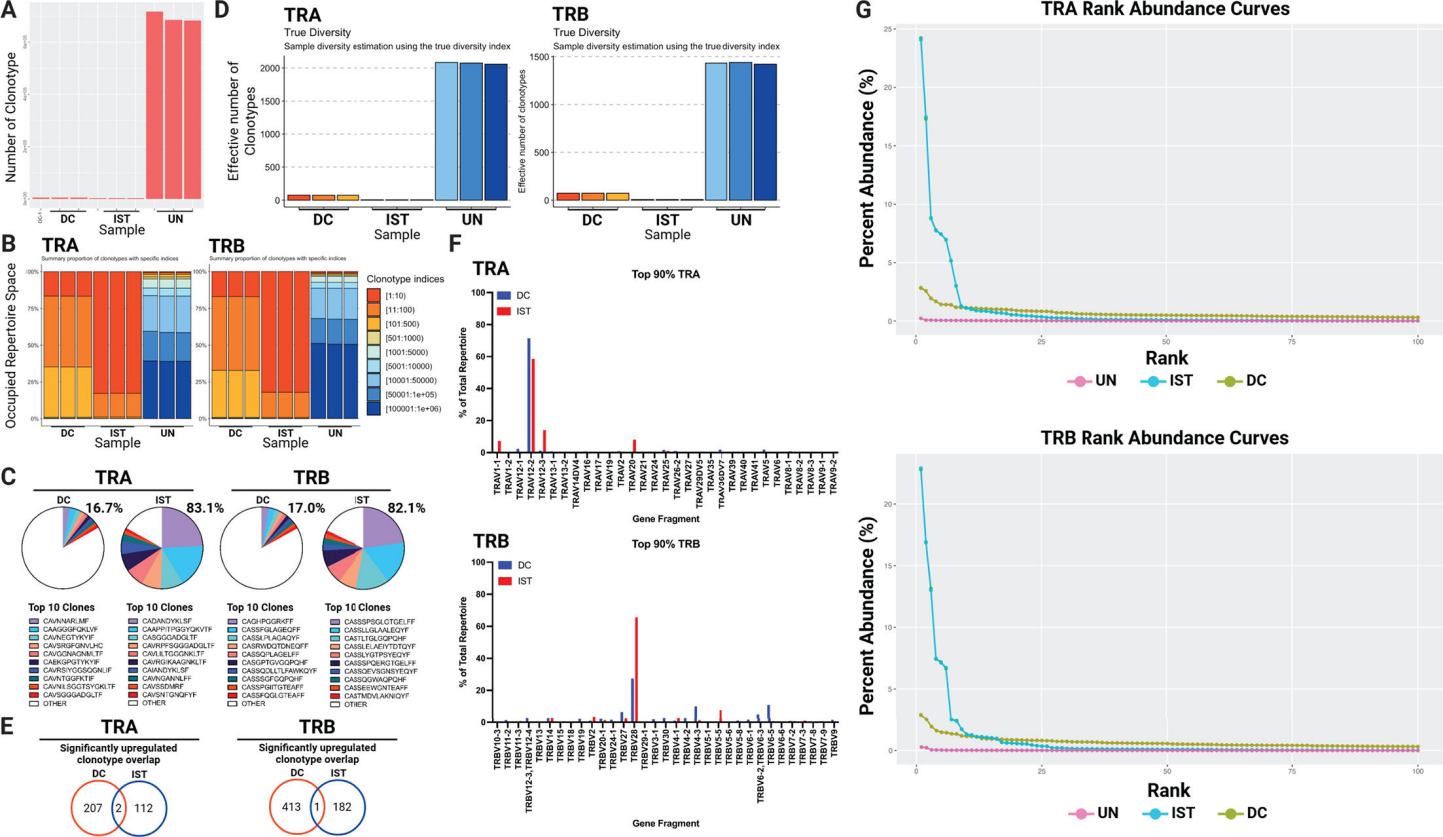
Treatment of naive CD8+ T cells by MART-1-IST yields a focused TCR repertoire. (**A**) Bar plot showing the total number of unique clonotypes detected in each sample. Each bar represents a sample, with the height indicating the total number of clonotypes present. Each sample was run in technical triplicate as shown. (**B**) Stacked bar charts representing the summary proportion of clonotypes with specific indices for TRA and TRB. Each bar represents one sample, with each sample run in technical triplicate, with the percentage of occupied repertoire space taken up by each stacked bar within each sample. Each color denotes a different clonotype index range, highlighting the distribution and diversity of clonotypes in each sample. (**C**) Pie charts representing the top 10 clones in DC- vs IST-treated samples. Pie charts show the repertoire space taken up by each of the top 10 identified clones based on the CDR3 region, as indicated for both TRA and TRB, with total percentage taken up by the top 10 clones shown. (**D**) True diversity index estimation of clonotype diversity for TRA and TRB across samples. The height of each bar represents the effective number of clonotypes, illustrating the diversity within each sample, accounting for richness and evenness in the samples. (**E**) Venn diagram of overlapping significantly upregulated clonotypes based on CDR3 sequencing in DC vs IST samples. Venn diagrams were generated based on the number of significantly upregulated clonotypes compared to untreated in both DC- and IST-stimulated samples. CDR3 regions that appeared as upregulated in both samples take up the middle section. (**F**) Alpha and beta TCR gene usage of the top 90% clones for each sample. Each plot shows the frequency of specific gene segments, comparing the gene usage between IST and DC stimulation. (**G**) Rank-abundance curves for TRA (top) and TRB (bottom) showing percentage abundance for TCR ranks up to 100 in MART-1-IST or MART-1-DC stimulated or untreated samples in donor HGLK0122.

We used TCR immunoprofiling (DriverMap Adaptive Immune Receptor Repertoire Profiling Assay) to compare clonal diversity in MART-1-specific CD8+ T cells expanded by αCD28-MART-1-IST or MART-1-mDC. This assay combines multiplex RT-PCR and next-generation sequencing to analyze TCR alpha (TRA) and beta (TRB) chain sequences and CDR3 regions. Sorted MART-1-specific CD8+ T cells from donor HGLK0086 and unsorted samples from donor HGLK0122 were analyzed, with untreated naive CD8+ T cells serving as controls (Fig. 5; Fig. S5).

Repertoire richness analysis (43) (Fig. 5A) showed markedly fewer unique TCR sequences in MART-1-specific cells expanded by either αCD28-MART-1-IST (2,935 clones) or MART-1-mDC (5,274 clones) compared to untreated naive cells (694,822 clones). Repertoire evenness analysis (Fig. 5B) indicated that αCD28-MART-1-IST-expanded cells were less diverse, with the top 10 TRA and TRB clonotypes occupying ~83% of the repertoire, compared to ~17% in MART-1-mDC-expanded cells. A breakdown of the CDR3 regions of the top 10 clones for DC and IST is shown in Fig. 5C. Furthermore, true diversity indices were obtained (44), which refers to the number of equally abundant types needed for the average proportional abundance of the types to equal that observed in the data set of interest where all types may not be equally abundant, calculated as the inverse of the weighted generalized mean of order *q* of the proportion (*p*) of each species within a population of size *N* (45). True diversity analysis again confirmed lower clonotype diversity in αCD28-MART-1-IST-expanded cells (Fig. 5D). Bulk analysis of donor HGLK0122 revealed similar trends, though with higher background TCR sequences (Fig. S5D). Venn diagrams of TCRA and TCRB CDR3 sequences revealed minimal overlap (one to two shared sequences) between MART-1-specific clonotypes expanded by αCD28-MART-1-IST and MART-1-mDC, consistent with the high diversity of naive TCR repertoires (Fig. 5E; Fig. S5E). We also assessed gene usage as shown in Fig. 5F, where both methods showed a preference for TRAV12-2, a segment commonly associated with MART-1/MELAN-A-reactive T cells in HLA-A2 donors (46–49). TRBV28 was prominent in cells expanded by both methods, but TRBV4-3 and TRBV6-5 were more frequent after MART-1-mDC treatment, while TRBV5-5 was preferred in αCD28-MART-1-IST-expanded cells. These results demonstrate that while both αCD28-MART-1-IST and MART-1-mDC expand diverse clonotypes, MART-1-mDC stimulates a broader array of MART-1-specific TCRs. This pattern was consistent across two donors. Finally, to further validate our findings regarding richness and evenness, we generated rank-abundance curves for both TRA and TRB repertoires (Fig. 5G), capping the displayed rank at 100 due to the negligible abundance of lower-ranked clonotypes. These curves highlight the marked expansion of a limited number of clones in the IST conditions, consistent with the observed reduction in TCR diversity within these samples.

### Stimulation by αCD28-SL9-IST but not SL9 peptide-loaded mDC expands naive HIV-specific CD8+ T cells in multiple HIV seronegative donors

Selective activation and expansion of T cells specific for conserved viral epitopes may facilitate the control of chronic infections such as HIV. In a proof-of-concept experiment, we assessed whether naive SL9-specific CD8+ T cells targeting the HLA-A*02:01-restricted HIV-1 p17 Gag epitope (SLYNTVATL; SL9) could be expanded using IST constructs. We compared FLAG-SL9-IST (MODless, TCR signal only), αCD28-SL9-IST (TCR + CD28 costimulatory signals), and SL9-peptide-loaded mDCs.

**Fig 6:**
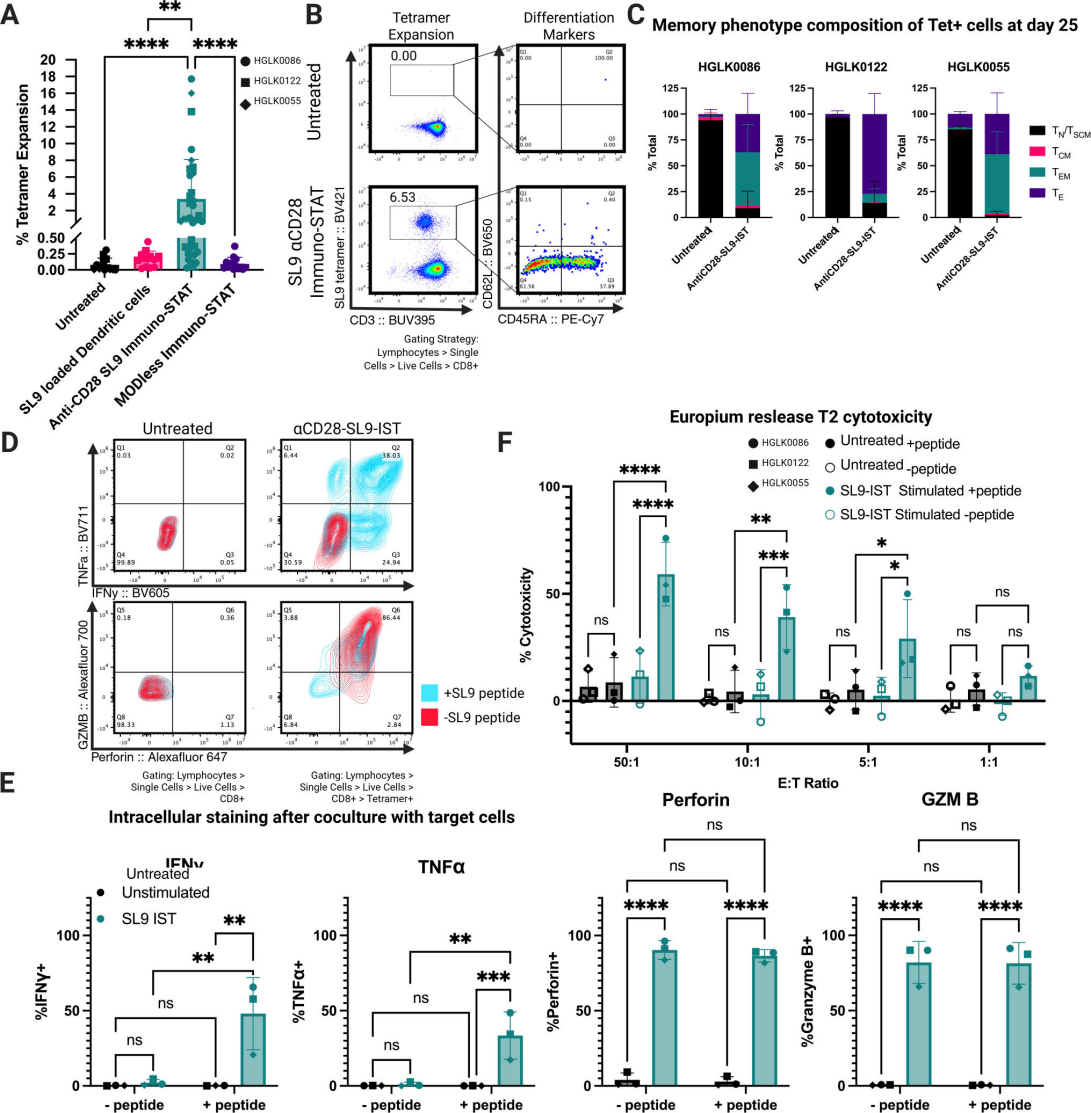
IST treatment of naive CD8+ T cells yields highly functional differentiated cells against the HIV-derived SL9 antigen in multiple donors without HIV. (**A**) Individual expansion data for SL9. Tetramer positivity detected by flow cytometry after 25 days in culture after stimulation with either SL9-loaded mDC, αCD28-SL9-IST, FLAG-SL9-IST, or left untreated and cultured with the IL21/7/15 cytokine mix. Successful replicates were determined by a cutoff of expansion greater than or equal to 0.5% of total CD8+ T cells. Individual data points represent individual biological replicates (*n* = 9–29) from multiple donors (*n* = 3) done in separate experimental replicates (two to five per donor) shown as different shapes. Significance was calculated in GraphPad Prism 10.4.0 on Kruskal-Wallis test (*P < 0.05, **P < 0.01, ***P < 0.001, and ****P < 0.0001). (**B**) Representative flow cytometry plots of phenotype gating strategy. Tetramer-positive cells were stratified by their expression of CD62L and CD45RA. (**C**) Memory phenotype composition analysis for three donors on day 25. Stacked bar plot shows the memory composition of tetramer-positive cells on day 25 after treatment with 100 nM αCD28-SL9-IST or untreated. Due to the lack of events to gate on for tetramer+ cells in untreated samples, stacked bar graphs were generated from total CD8+ T cells for untreated samples and from tetramer+ cells of successful replicates for IST stimulated (%tet+ > 0.05), *n* = 5–12 biological replicates for each donor (*n* = 3). (**D**) Representative flow plots for intracellular cytokine staining. Contour plots show tetramer-positive cells stimulation by either SL9 peptide-loaded T2 cells (blue) or vehicle control (red). Contour plots assess dual expression of interferon-gamma and TNF-alpha (top) or perforin and granzyme B (bottom). Contour plots were gated on tetramer-positive cells for αCD28-SL9-IST-treated samples and total CD8+ cells for untreated samples. (**E**) Intracellular staining. Bar plots showing data for intracellular staining for cytokine expression. Each data point represents results averaged from *n* = 3 biological replicates from *n* = 3 donors, with experiments from each donor conducted on separate days. Expression was determined on tetramer-positive cells for αCD28-SL9-IST-treated samples and total CD8+ cells for untreated samples. (**F**) Europium release cytotoxicity assay. Summary results from europium-based release assay against peptide-loaded or vehicle control-loaded T2 cells by untreated or αCD28-SL9-IST-treated cells. Each data point represents averaged results from three biological replicates from *n* = 3 donors. All statistical analysis was done in GraphPad Prism 10.4.0. Significance was estimated by a two-way ANOVA, and group differences at each ratio were computed and assessed via analyses of simple effects, using the error term and degrees of freedom from the whole design (*P < 0.05, **P < 0.01, ***P < 0.001, and ****P < 0.0001).

Unlike MART-1-specific CD8+ T cells, which were effectively expanded by MART-1-peptide-loaded mDCs, SL9-peptide-loaded mDCs failed to expand naive SL9-specific CD8+ T cells in three HIV-negative donors across multiple replicates (Fig. 6A). However, αCD28-SL9-IST treatment successfully expanded SL9-specific CD8+ T cells to detectable levels (>0.5%) in all three donors using the IL21/7/15 cytokine protocol. Conversely, FLAG-SL9-IST treatment (TCR signal only) did not expand SL9-specific CD8+ T cells in any donor. The identity of expanded SL9-specific cells was confirmed by dual-tetramer staining with SL9 tetramers conjugated to PE and BV421 (Fig. S3C). Expansion ranged from 0.5% to 18% of total CD8+ cells in positive biological replicates.

While αCD28-MART-1-IST expanded MART-1-specific CD8+ T cells in all replicate wells, SL9-specific CD8+ T cell expansion by αCD28-SL9-IST occurred in subsets of replicate wells: HGLK0086 (6/9), HGLK0122 (7/12), and HGLK055 (3/5). This reduced frequency of expansion in replicate wells likely reflects the ~65-fold lower precursor frequency of SL9-specific naive CD8+ T cells (~1 in 500,000) compared to MART-1-specific naive CD8+ T cells (~1 in 7,500) (35).

SL9-specific CD8+ T cells expanded by αCD28-SL9-IST displayed a mix of TE and TEM phenotypes, as shown in representative dot plots (Fig. 6B) and summary data from three donors (Fig. 6C). In contrast, untreated naive CD8+ T cells maintained a naive-like phenotype across all donors. These results parallel findings for MART-1-specific CD8+ T cells expanded by αCD28-MART-1-IST, suggesting a consistent effect of IST treatment across antigens, though further studies with additional antigens are needed to confirm this hypothesis.

Polyfunctionality of αCD28-SL9-IST-expanded CD8+ T cells was assessed by IFNγ, TNFα, perforin, and granzyme B production after a 16-hour coculture with SL9-peptide-loaded or unloaded T2 cells. These cells exhibited high baseline perforin and granzyme B levels, with significant increases in IFNγ production after stimulation with SL9-peptide-loaded T2 cells (*P* < 0.01) (Fig. 6D and E). Notably, TNFα production was observed in αCD28-SL9-IST-expanded CD8+ T cells upon SL9-peptide stimulation, contrasting with the lack of TNFα production in MART-1-specific CD8+ T cells expanded by αCD28-MART-1-IST under similar conditions. Cytotoxicity was evaluated using a europium release assay (Fig. 6F). αCD28-SL9-IST-expanded CD8+ T cells demonstrated significant killing of SL9-peptide-loaded T2 cells at 50:1 (*P* < 0.0001), 10:1 (*P* < 0.001), and 5:1 (*P* < 0.05) E:T ratios, but no significant activity at 1:1. Variability in cytotoxicity likely reflects differences in the fraction of SL9 tetramer+ CD8 T cells across donors (Fig. S3D). These findings demonstrate that αCD28-SL9-IST effectively activates and expands SL9-specific CD8+ T cells from the naive repertoire of multiple HIV-negative donors, achieving phenotypic and functional outcomes not observed with SL9-peptide-loaded mDCs.

### αCD28-SL9-IST-treated SL9-specific cells also have a differentiated epigenetic methylation

**Fig 7:**
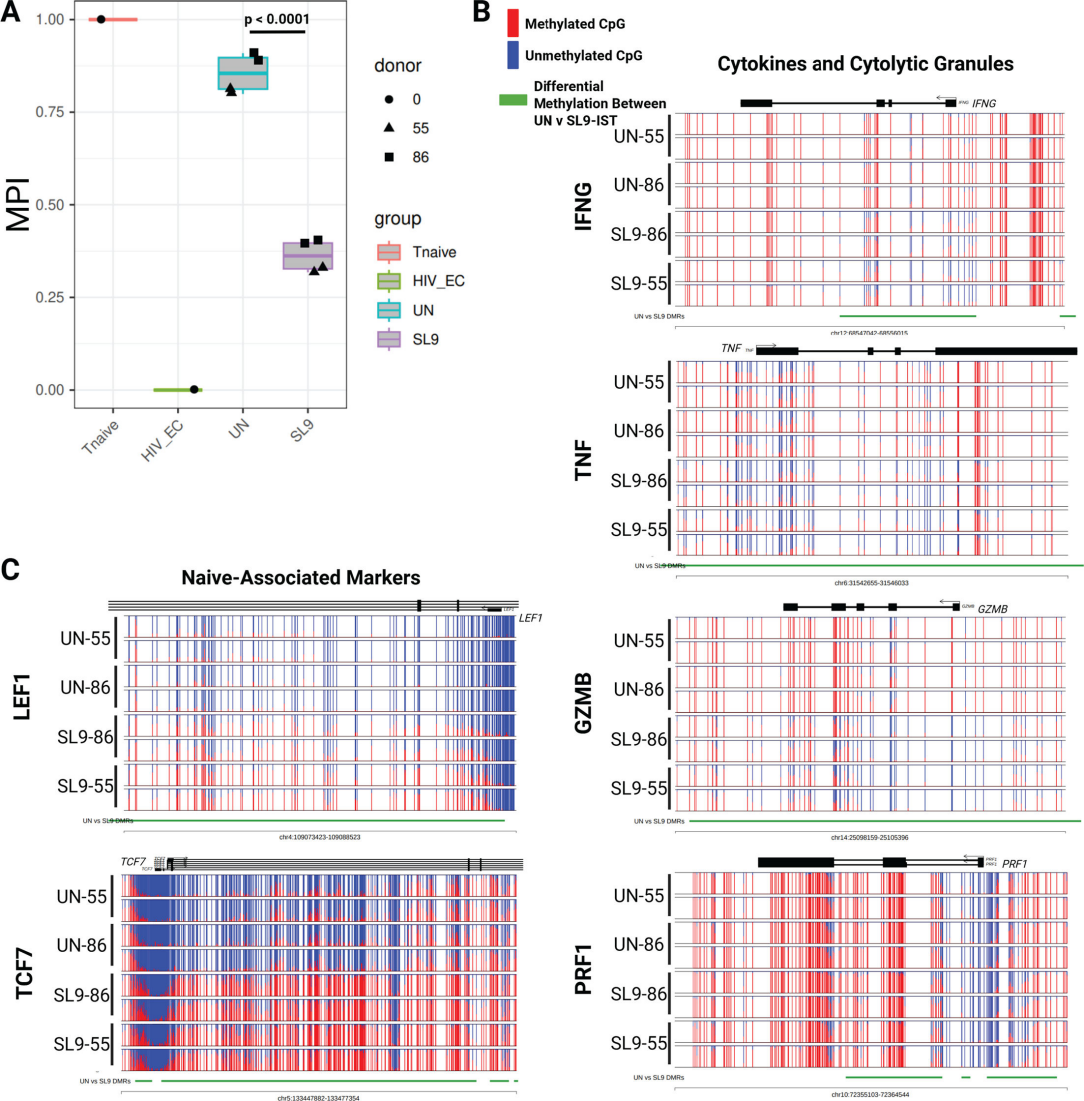
Epigenetic signatures of SL9-IST treatment are consistent with differentiated cells. (**A**) T cell multipotency index. T cell multipotency score was generated from 0 to 1 for each indicated sample (UN, untreated; SL9, SL9 Immuno-STAT treated) and compared to true naive (1) and terminally exhausted (0) reference samples. Each dot represents one technical replicate from two donors shown by different shapes. (**B**) Normalized plots of CpG methylation at sites surrounding and within DMRs of effector molecules (IFNG, TNF, GZMB, and PRF1) and (**C**) naive associated transcription factors (LEF1 and TCF7) obtained from WGBS EM Seq analysis. Red and blue lines depict methylated and unmethylated CpG sites, respectively, while green lines depict differentially methylated regions between SL9 and UN. Significance was calculated by ordinary one-way ANOVA, followed by Tukey’s multiple comparisons test using GraphPad Prism 10.4.0.

We performed whole-genome methylation profiling (EM Seq) on bulk unstimulated naive CD8+ T cells and highly purified SL9-specific CD8+ T cells expanded by αCD28-SL9-IST from two donors to assess differentiation-associated programs using the multipotency index (Fig. 7A). The multipotency index of SL9-specific CD8+ T cells expanded by αCD28-SL9-IST was 0.40 (HGLK0086) and 0.32 (HGLK0055), comparable to that observed for MART-1-specific CD8+ T cells expanded by αCD28-MART-1-IST (0.35 and 0.37 for HGLK0055 and HGLK00122, respectively). Pairwise comparison of differentially methylated regions identified 78,190 DMRs with >20% methylation differences between untreated and αCD28-SL9-IST-expanded SL9-specific CD8+ T cells (*n* = 2 donors). Similar to MART-1-specific CD8+ T cells, SL9-specific CD8+ T cells expanded by αCD28-SL9-IST exhibited reduced methylation at loci encoding IFNG, TNF, GZMB, and PRF1, consistent with their enhanced cytotoxic and functional activity (Fig. 7B). Additionally, both donors showed increased methylation at loci associated with stem cell factors TCF7 and LEF1, reflecting a more differentiated phenotype (Fig. 7C). These findings demonstrate consistent epigenetic changes in CD8+ T cells expanded by αCD28-SL9-IST and αCD28-MART-1-IST, characterized by increased methylation of naive/stem cell-associated markers and demethylation of cytotoxic functional genes.

### αCD28-SL9-IST-treated SL9-specific cells display an oligoclonal response in multiple donors as determined by TCR immunoprofiling

**Fig 8:**
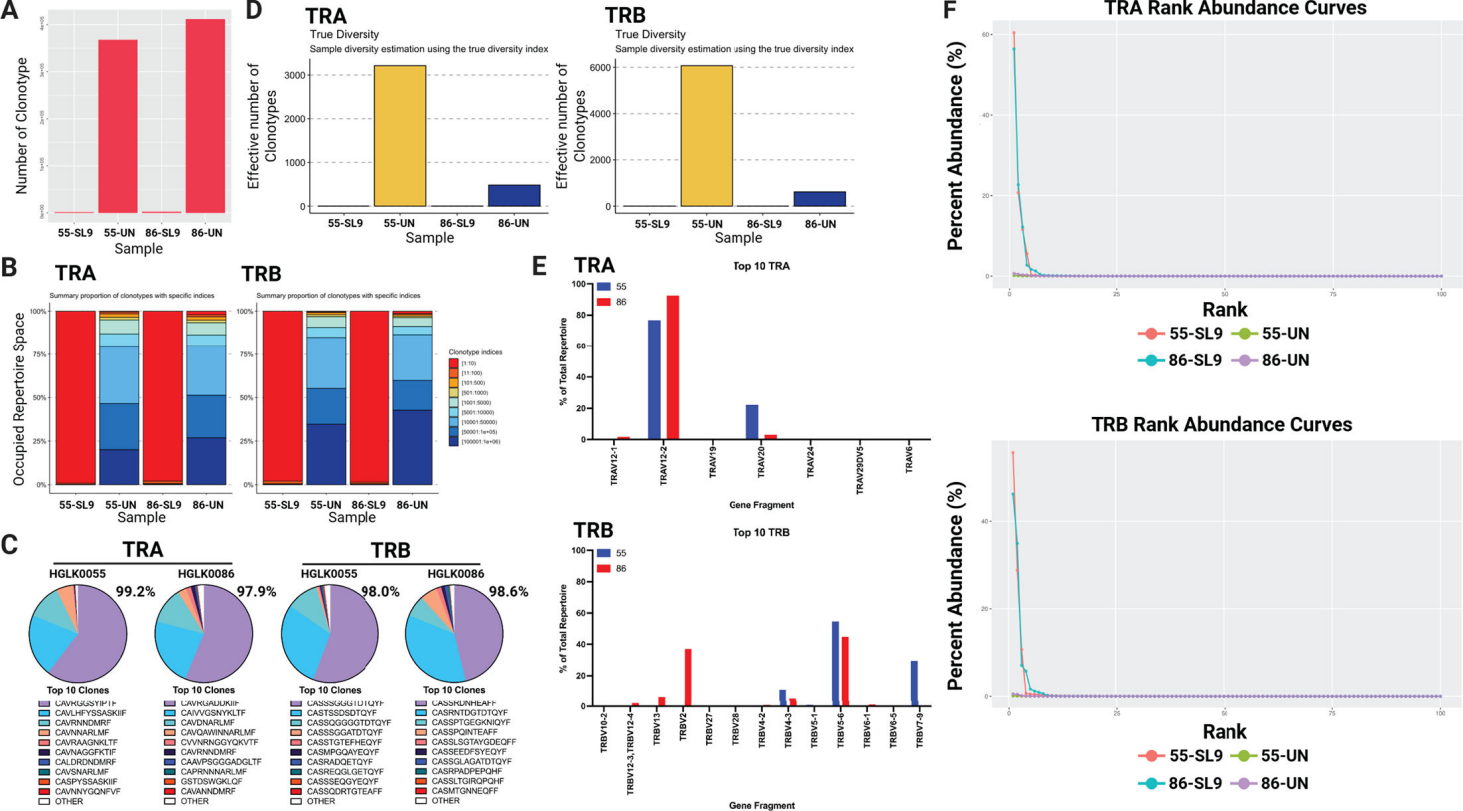
Treatment of naive CD8+ T cells by SL9-IST yields a focused TCR repertoire. (**A**) Bar plot showing the total number of unique clonotypes detected in each sample. Each bar represents one sample, with the height indicating the total number of unique clonotypes identified by sequencing CDR3 regions. (**B**) Stacked bar charts representing the summary proportions of clonotypes with specific indices for TRA and TRB. Each bar represents one sample, with the percentage of occupied repertoire space taken up by each stacked bar within each sample. Each color denotes a different clonotype index range, highlighting the distribution and diversity of clonotypes in each sample. (**C**) Pie charts showing repertoire space taken by the top 10 identified clonotypes for TRA and TRB for each donor. The top 10 clones based on CDR3 sequencing for both TRA and TRB for both donors are plotted, showing occupied repertoire space for each individual clone. The percentage occupied by the top 10 clonotypes is indicated next to each pie chart. (**D**) True diversity index estimation of clonotype diversity for TRA and TRB across samples. The height of each bar represents the effective number of clonotypes based on true diversity calculation. (**E**) Alpha and beta TCR gene usage of the top 10 clones for each sample. Each plot shows the frequency of specific gene segments, comparing the gene usage between IST stimulation from the two donors. (**F**) Rank-abundance curves for TRA (top) and TRB (bottom) showing percentage abundance for TCR ranks up to 100 in SL9-IST stimulated or untreated samples in donors HGLK0055 and HGLK0086.

To examine clonotype diversity of SL9-specific CD8+ T cells expanded by αCD28-SL9-IST, we performed TCR immunoprofiling on SL9-specific CD8+ T cells isolated 25 days after treatment in donors HGLK0055 and HGLK0086. Similar to MART-1 findings, SL9-specific CD8+ T cells showed a marked reduction in unique TCR clonotypes compared to unsorted T cells (Fig. 8A). Repertoire evenness analysis revealed that nearly 100% of the SL9-specific clonotype repertoire was dominated by fewer than 10 clones (Fig. 8B). In donor HGLK0086, approximately six clonal sequences comprised ~98% of the TRA and TRB repertoire, with the top clone accounting for 56.4% (TRA) and 46.2% (TRB) of the total. For donor HGLK0055, approximately three to four clones occupied ~98% of the repertoire, with the top clone representing 60.5% (TRA) and 55.7% (TRB) (Fig. 8C). This limited diversity may be due to the lower precursor frequency of SL9-specific naive CD8+ T cells compared to MART-1-reactive cells. True diversity indices confirmed reduced diversity in SL9-specific CD8+ T cells expanded by αCD28-SL9-IST compared to untreated samples (Fig. 8D). Gene usage analysis showed a strong bias toward the TRAV12-2 gene, associated with HLA-A2*02:01 binding (Fig. 8E). The most common TRBV segment in both donors was TRBV5-6, but the second most common segment differed, with TRBV2 in HGLK0086 and TRBV7-9 in HGLK0055, as shown in Fig. 8F. Finally, to further validate our findings regarding richness and evenness, we generated rank-abundance curves for both TRA and TRB repertoires (Fig. 8G), displaying the top 100 clonotypes due to the negligible abundance of lower-ranked clones. These curves demonstrate consistent expansion of a limited number of clones in the SL9-IST conditions across both donors, supporting the broader applicability of these observations. These findings indicate that αCD28-SL9-IST treatment elicits an oligoclonal response in SL9-specific CD8+ T cells, using similar but donor-unique gene fragments in healthy individuals.

## DISCUSSION

In the current study, we demonstrate that Immuno-STAT, delivering TCR and CD28 costimulatory signals, facilitates robust *ex vivo* priming, expansion, and differentiation of primary human antigen-specific naive CD8+ T cells. Notably, αCD28-SL9-IST is the first artificial APC-based therapy to robustly expand SL9-specific CD8+ T cells from donors without HIV infection. While soluble pMHC molecules have been used to amplify bulk (50) or memory CD8+ T cells (28–30), IST is the first fully protein-based, soluble c-pMHC-costimulatory molecule capable of priming and markedly expanding naive antigen-specific CD8+ T cells *ex vivo*. This study, using highly purified naive CD8+ donor T cells, provides detailed functional, phenotypic, and genetic analyses, comparing αCD28-IST-expanded cells to those expanded by autologous mDCs, and validates the indispensable role of the αCD28 costimulatory domain for effective naive CD8+ T cell priming and expansion.

Previous studies from our lab showed that MODless IST constructs expand memory SL9- and CMV-reactive CD8+ T cells, generating highly functional cells with potent anti-HIV and anti-CMV properties both *ex vivo* and *in vivo* from seropositive individuals (30). These findings confirmed that IST delivering only a TCR signal suffices for memory CD8+ T cell activation and proliferation, consistent with reports that costimulatory signals are unnecessary for memory T cell reactivation and expansion (51). However, MART-1 or SL9 IST constructs lacking a costimulatory domain failed to expand naive MART-1 or SL9-specific CD8+ T cells, respectively, even at high concentrations. In contrast, αCD28-IST constructs presenting MART-1 or SL9 peptides robustly activated and expanded naive MART-1 or SL9-specific CD8+ T cells, respectively, confirming that costimulatory signals, such as those provided by CD28, are required for naive CD8+ T cell activation (10, 52). Interestingly, while subpicomolar concentrations (0.01–0.1 pM) of IST efficiently expanded memory SL9- and NLV-specific CD8+ T cells in earlier studies (30), concentrations of 50 nM or higher were required for significant MART-1-specific naive CD8+ T cell expansion. This requirement for higher concentrations of IST to activate naive T cells than memory T cells is likely due to the greater sensitivity to cognate antigens of TCRs expressed by memory T cells compared to naive T cells (53–55). While a prior study reported *ex vivo* expansion of MART-1-specific CD8+ T cells by treatment with a MART-1 IST linked to IL-2 (29), that study used unfractionated PBMCs, which include effector and central memory CD8+ T cells that do not require costimulatory signals and may have been the source of the expanded T cells. In contrast, our study used highly purified naive CD8+ T cells to definitively demonstrate MART-1- and SL9-specific T cell expansion from the naive T cell repertoire. These results underscore the critical requirement for combining TCR and costimulatory signals in aAPC-based therapies targeting expansion of the naive CD8+ T cell repertoire.

Interestingly, while we observed that both DC- and IST-expanded MART-1-specific CD8+ T cells were polyfunctional after peptide re-exposure, IST-expanded cells expressed perforin and granzyme B even at baseline without peptide during the ICS assay. We hypothesize that these cytotoxic molecules are imprinted during initial priming by the strong TCR and CD28 signaling delivered by the IST expansion, which drives a late effector-memory state with constitutive perforin and granzyme B expression, stored in granules for immediate use as described (56). Thus, their baseline expression reflects high resting cytotoxic potential rather than acute activation. This is supported by the 3-hour T2 cytotoxicity assay, where IST-expanded cells consistently outperformed DC-expanded cells across donors. While αCD28-IST expanded MART-1-specific CD8+ T cells in naive CD8+ T cells from all donors tested, MART-1-mDC cultures resulted in faster and significantly greater expansion (*P* < 0.01) compared to αCD28-MART-1-IST-treated CD8+ T cells. This is likely a consequence of the robust functionality of mDCs due to their expression of diverse costimulatory and adhesion molecules, including OX-40L, 41BB, and LFA-1 (57, 58), which may enhance expansion kinetics and broaden precursor TCR clonotype activation. We do not know why ISTs, but not mDCs, were able to expand naive SL9-specific CD8+ T cells, while mDCs induced greater expansion of MART-1-specific T cells than IST treatment. It is possible that naive antigen-specific T cells present at high precursor frequencies respond differently to mDC activation than naive antigen-specific T cells present at low precursor frequencies. Additionally, while IST restricts activation to CD8+ T cells specific to the cognate antigen they are presenting, mDCs express a wide range of MHC, costimulatory, and adhesion molecules, which may activate bystander cells and adversely affect the expansion of low precursor frequency CD8+ T cells by competing with them for cytokines and other growth factors. This may be related to the late emergence of SL9 responses after infection with SL9-specific CD8+ T cell responses absent during acute infection and the dominant HLA-A*0201-restricted CD8+ T cell response during chronic infection (59). In future studies, we will explore the role of precursor frequency on antigen-specific CD8+ T cell expansion by mDC and IST by comparing their ability to expand high and low precursor frequency CD8+ T cells specific for other viral epitopes. Nevertheless, the delivery of only TCR and CD28 costimulatory signals by αCD28-IST effectively expanded naive SL9-specific CD8+ T cells, supporting its promise as a potential HIV immunotherapeutic for further optimization. Rapid advancement of HIV-specific ISTs into clinical trials will be supported by the excellent safety profile of IST constructs incorporating affinity-attenuated IL-2 (28, 29), which are under clinical evaluation for recurrent metastatic HPV-positive head and neck squamous cell carcinoma and WT1+ metastatic solid cancers (NCT03978689/NCT04852328 and NCT05360680).

We initially evaluated the capacity of IST to activate and expand MART-1-specific naive T cells due to their high precursor frequency in healthy populations (~1/7,500) (34, 35) and frequent use in validating aAPC platforms (16, 21, 24, 36, 60–64). To evaluate the potential of ISTs as an immunotherapeutic to treat HIV, we tested its ability to activate and expand naive CD8+ T cells specific for the HLA-A*02:01-restricted HIV p17 Gag-derived epitope SLYNTVATL (SL9), which notably has a much lower precursor frequency (~1/500,000) (35). Although their prevalence is low in the acute stages of HIV acquisition, the levels of SL9-specific CD8+ T cells subsequently rise during chronic infection, with up to 75% of HLA-A*02:01 individuals eventually generating anti-SL9 CD8+ T cell responses (59, 65). However, while an inverse correlation with viral loads and the magnitude of HLA-A and HLA-B-restricted Gag-specific epitope responses in progressive infection has been reported (59, 66–68), effective control of viremia is more significantly correlated with HLA-B-restricted Gag responses than HLA-A-restricted Gag-specific responses (68, 69). For this reason, HIV-specific CD8+ T cells are promising targets for adoptive transfer or expansion by vaccine strategies, particularly soon after HIV infection (70–75). To generate CD8+ T cells with more potent anti-HIV activity, we will leverage the modular design of the IST platform to swap out the MHC and peptide domains to express either escape mutant SL9 peptides or HLA-B-restricted HIV epitopes.

Previous efforts using peptide-loaded mature dendritic cells or other aAPC platforms have failed to expand detectable levels of SL9-specific CD8+ T cells in HIV-negative individuals (71). Similarly, while SL9-reactive T cell clones demonstrated potent *ex vivo* HIV suppression, their adoptive transfer has not yielded clinical success (76, 77). Consistent with prior reports, our mDC priming system failed to expand SL9-specific CD8+ T cells in any donor tested, possibly due to the use of mature rather than immature DCs (78). In contrast, αCD28-SL9-IST substantially expanded SL9-specific CD8+ T cells from naive populations in multiple HIV-negative donors, achieving up to 18% of the total CD8+ T cell population. These cells exhibited high cytotoxicity in peptide-loaded T2 europium release assays and produced IFN-γ, TNF-α, perforin, and granzyme B, key attributes for effective HIV control (79, 80). We plan to compare their avidities to those of naturally generated SL9-specific CD8+ T cells in PWH. This is the first report of an *ex vivo* strategy to expand detectable oligoclonal SL9-specific CD8+ T cell populations in HIV-negative individuals using an aAPC platform, marking a significant advancement in HIV immunotherapy. While the ability of IST to generate a response toward SL9 may be beneficial in the acute phase of HIV infection, this response may be compromised by the eventual emergence of immune escape mutants (65). Unlike previous monoclonal TCR-engineered approaches (77, 81, 82), which are limited by immune escape mutants, IST has the potential to expand diverse clonotypes, enabling potential redirection of the immune response in HIV patients to target antigens, most importantly to include SL9 escape variants. Future studies will investigate the capacity of a cocktail of αCD28-ISTs specific for the major SL9 escape mutants, SLYNTIAVL(V82I/T84V), SLFNTVATL(Y79F), and SLFNTIAVL (Y79F/V82I/T84V), to activate and expand SL9-variant specific CD8+ T cells to eliminate the predicted immune escape variants (83–85). Of note, a potential benefit of constructing αCD28-ISTs that activate and expand CD8+ T cells that recognize the SL9 variant SLFNTIAVL is the breadth of SLFNTIAVL-specific CD8+ T cell recognition, which includes SL9 and other immune escape SL9 variants, enabling them to eliminate cells infected with immune escape variants (86). We could also generate ISTs to activate and expand naive CD8+ T cells specific for HIV-derived epitopes that are highly conserved and immunoprotective, such as WIILGLNKIVR. This epitope is part of a conserved element identified within Gag p24, containing exclusively amino acids that are at least 98% sequence conserved across all group M sequences (87). It may be a preferential target in HIV-1 controllers (88).

Our findings highlight the potential of the IST platform to generate robust anti-HIV CD8+ T cells from the naive repertoire, supporting its use in ACT against conserved antigens or escape variants to which the immune system has yet to mount a response. However, clinical trials show improved and sustained outcomes with adoptive transfer of less differentiated cells (89). Although IST-derived MART-1 and SL9-specific CD8^+^ T cells displayed potent effector function and cytotoxic molecule expression, IST-generated CD8+ T cells exhibited a higher degree of differentiation than those generated by mDCs, as indicated by memory phenotype and epigenetic analysis. For HIV, where persistent antigen is sparse and immune escape variants can arise, generating a population of less differentiated, multipotent memory cells (especially T_SCM_ and T_CM_) may be essential to ensure persistent T cell responses to suppress the initiation of systemic infection after viral reactivation. Therefore, we will plan to investigate strategies to modulate the strength or duration of stimulation, optimize costimulatory signals, and select cytokines to promote a higher fraction of stem-like or central memory cells while optimizing the balance between effector function and long-term persistence. Future studies will investigate strategies to generate less differentiated cells by modifying IST design, including alternate costimulatory domains, such as CD27 ligands, antigen affinities, cytokine signaling domains (such as IL-7, IL-15, or IL-21), and dosing regimens (90–93).

αCD28-IST represents a proof-of-concept off-the-shelf platform for the *ex vivo* expansion of HIV-specific CD8+ T cells from the naive repertoire, offering a targeted approach to generate precise immune responses against selected antigens to which the immune system has not yet mounted a response. This study underscores its transformative potential for HIV immunotherapy by demonstrating its ability to overcome barriers associated with expanding CD8+ T cells targeting challenging antigens, such as HIV Gag-derived SL9. By enabling robust and scalable generation of clinical-grade T cells, IST could address the limitations of conventional methods in adoptive cell transfer. The modular design of IST allows for customization with diverse peptides, HLA alleles, and costimulatory ligands, enabling tailored immune responses to less immunogenic but clinically significant targets. This adaptability is particularly relevant for HIV, where generating durable and effective antigen-specific T cells remains a critical challenge. We hypothesize that the optimum time to stimulate HIV-specific CD8+ T cell responses with αCD28-IST treatment would be early in the course of infection during ART, when antigen levels are limited but not fully absent. Administering αCD28-IST in this lower antigen, lower inflammatory environment would allow for the priming of highly functional, non-exhausted HIV-specific CD8^+^ T cells that can differentiate into durable effector-memory populations, positioning them to respond rapidly if viral reactivation occurs.

Future investigations will explore IST’s potential for *in vivo* naive T cell expansion and its viability as a CD8+ T cell vaccine platform, further advancing its application in HIV, other infectious diseases, and cancer. By providing a scalable, versatile solution for amplifying virus- and cancer-specific CD8+ T cells, αCD28-IST has the potential to revolutionize immunotherapy, paving the way for innovative treatments for HIV, other persistent infections, and cancer.

## MATERIALS AND METHODS

### Expression and purification of Immuno-STAT proteins

Immuno-STAT proteins (previously referred to as synTac proteins) were expressed, purified, and validated as previously described (30). Briefly, the MART-1 MODless IST (i.e., lacking a comodulatory module) consists of a heavy chain composed of HLA-A*0201 fused C-terminally to an IgG Fc domain with a N297Q mutation to abrogate FcR-mediated binding and signaling, and a light chain composed of β2M linked N-terminally to the MART-1 high-affinity peptide analog (26–35, ELAGIGILTV), with a disulfide bond engineered between the β2M and the MHC heavy chain (R12C-A236C). For the MART-1 anti-CD28 IST, the anti-CD28 scFv (Clone 9.3) (94) is linked to the N-terminus of the HLA-A*0201. ISTs were produced via transient transfection using the ExpiCHO expression system (Gibco). Culture supernatant, harvested 14 days post-transfection, was clarified by centrifugation and purified on a HiTrap mAbSelect SuRe column (Cytiva) using an ÄKTA Xpress FPLC (GE HealthCare). Proteins were eluted with a linear gradient of 1.5 M ArgCl (pH 6.5–3.5) over 10 column volumes, with peak fractions neutralized with 1.5 M ArgCl-10 mM Tris (pH 8.8), pooled, and sterile filtered. Further purification was performed by gel filtration chromatography (Superdex S200 26/60, GE HealthCare) in PBS with 0.5 M NaCl. Buffers were endotoxin-free, and columns were pre-cleaned with 0.5 M NaOH. Endotoxin levels were tested using the Lonza Kinetic-QCLTM Chromogenic LAL Assay Kit.

### Isolation of PBMC

Donor PBMCs were isolated from leukapheresis products using an Einstein IRB-approved protocol of the Einstein-Rockefeller-CUNY Center for AIDS Research (ERC-CFAR). Briefly, 5 mL of leukapheresis product diluted in 25 mL of 1× PBS was overlaid on 14 mL of Ficoll-PaqueTM PLUS (Fisher, 45001750) in 50 mL conical tubes. Cells were centrifuged at 320 × *g* for 30 minutes with acceleration and deceleration set to 0. After centrifugation, the PBMC layer at the PBS and Ficoll-Paque PLUS interface in each tube was collected and transferred to a new 50 mL conical tube. An additional 1× PBS (up to 50 mL) was added, and cells were centrifuged again at 320 × *g* for 10 minutes with maximum acceleration and deceleration. The supernatant was aspirated, and the cell pellets were combined, counted, and resuspended at a maximum of 50 million cells per mL in FBS with 10% DMSO. PBMCs were slowly cooled (~1°C/minute) using an isopropanol freezing container (Mr. Frosty) and stored at −80°C overnight before transfer to −150°C for long-term storage.

### Cell lines

The T lymphoblast T2 (CRL-1992) cell line and SK-Mel-5 (HTB-70) human melanoma cell line were purchased from the American Type Culture Collection (ATCC). They were cultured in Iscove’s modified Dulbecco’s medium supplemented with 10% FBS, 1× GlutaMAX (Gibco, Ref# 35050-061), 1× penicillin-streptomycin solution (Corning, Ref# 30-002-CI), and 100 mM HEPES buffer at the cellular density recommended by the ATCC. All cell lines were maintained in a humidified 5% CO_2_ atmosphere at 37°C and regularly tested for mycoplasma contamination using MycostripTM (InvivoGen, rep-mys-10).

### Peptides

Synthetic peptides corresponding to the Melanoma-associated antigen recognized by T cells-1 (MART-1) 26-35 (Leu27; ELAGIGILTV) and the HIV-1-derived Gag 77-85 (SLYNTVATL) were obtained from GenScript. Peptides were dissolved in DMSO (10 mg/mL) and stored at −80^o^C before use.

### *In vitro* antigen-specific naive T cell stimulation

On day −1, PBMCs were thawed quickly in a 37°C water bath and washed twice with pre-warmed Iscove’s Modified Dulbecco’s medium (Corning, Ref# 10-016-CV) supplemented with 10% FBS, 1× GlutaMAX (Gibco, Ref# 35050-061), 1× Penicillin-Streptomycin Solution (Corning, Ref# 30-002-CI), and 100 mM HEPES buffer (I-10 media). On the same day, naive CD8+ cells were isolated by immunomagnetic sorting using the naive CD8+ T Cell Isolation Kit (Miltenyi Biotec, cat# 130-093-244) according to the manufacturer’s instructions and rested overnight in I-10 media supplemented with 5 ng/mL IL-7 (NIH) at 3 × 10^6^ cells per mL in a flask. Naive CD8+ T cell activation and expansion were performed as described (36). On day 0, cells were resuspended at 1 × 10^6^ cells/mL in I-10 media with 30 ng/mL IL-21, plated at 500,000 cells/well in 48-well plates, and stimulated with peptide-loaded mDCs (4:1 mDC:T ratio) or Immuno-STAT at specified concentrations for 72 hours. On day 3, cells were supplemented with fresh media containing IL-7 and IL-15 (5 ng/mL each, final concentrations). On day 7, cultures were transferred to 12-well plates and supplemented with additional media to maintain 5 ng/mL of each cytokine in a 2 mL volume. On day 10, cultures were transferred to 6-well plates, supplemented with additional media to maintain 10 ng/mL of each cytokine in a 3 mL volume. Media were exchanged every 2-3 days thereafter, maintaining 10 ng/mL of IL-7 and IL-15. For alternative cytokine conditions, cells were cultured with either IL-2 (100 U/mL) or IL-15 (10 ng/mL) at all time points (days 0, 3, 7, and beyond).

### Dendritic cell generation

mDCs utilized to stimulate naive CD8+ T cells were generated using the plastic adherence method and as previously described (36, 95). Briefly, monocytes were isolated via plastic adherence by incubating 10 × 10^6^ PBMCs in 3 mL in a 6-well plate for 2 hours. Non-adherent cells were removed by washing three times with PBS before adding 2 mL of fresh media supplemented with GM-CSF (800 U) (BioLegend, 713604) and IL-4 (800 U) (BioLegend, 574002). After 72 hours, 1.5 mL of fresh media supplemented with GM-CSF (1,600 U) and IL-4 (800 U) was added. After an additional 24 hours, the peptide of interest was added (2.5 µM), along with maturation factors IFN-γ (100 U) (BioLegend, 570202) and LPS (10 ng/µL) (Sigma, L6529). Following overnight incubation with maturation factors, mDCs were harvested and irradiated at 30 Gy before co-culture with isolated naive cells as described above. To confirm proper maturation, a sample of mDCs was analyzed by flow cytometric analysis after staining with antibodies to CD83 (BioLegend, 305316) and CD14 (BioLegend, 325606).

### *In vitro* cytotoxicity using the europium release assay

Cytotoxic activity of antigen-specific CD8+ T cells was assessed using DELFIA EuTDA Cytotoxicity Reagents (PerkinElmer, AD0116) with peptide-pulsed (10 µg/mL) or vehicle control (DMSO) pulsed T2 cells as target cells, following the manufacturer’s instructions. Cytotoxicity was assessed at a variety of E:T ratios (50:1, 10:1, 5:1, and 1:1) for 3 hours in the presence of probenecid (250 nM, Millipore Sigma, Cat #57-66-9). Percentage cytotoxicity was calculated for each replicate using the relationship: % cytotoxicity = (experimental release – spontaneous release)/(maximum release – spontaneous release) × 100%. Triplicate measurements were conducted for each condition, and triplicate values were averaged for each donor before statistical analysis.

### *In vitro* cytotoxicity using flow cytometry

For flow cytometry-based cytotoxicity assays, target cells were co-cultured with effector cells at the indicated E:T ratios and incubated overnight at 37°C in Iscove’s Modified Dulbecco’s medium (Corning, Ref# 10-016-CV) supplemented with 10% FBS, 1× GlutaMAX (Gibco, Ref# 35050-061), 1× Penicillin-Streptomycin Solution (Corning, Ref# 30-002-CI), and HEPES buffer (100 mM) without added cytokines. After 16 hours, the entire sample was collected and stained to quantify the remaining live target cells following the staining protocol outlined below. The total live target cell number was determined based on negative live/dead blue staining with effector cells excluded by gating on the CD3− CD8− population. Percentage cytotoxicity was calculated using the formula: % cytotoxicity = ([no effector control cell number − sample]/no effector control cell number) × 100%. Triplicates were conducted for each condition, and triplicate values were averaged for each donor before statistical analysis.

### Flow cytometry analysis of T cell phenotype

At each time point, after thorough resuspension of cells by pipetting, cells (300 µL) were harvested from each cell culture well and washed with PBS (200 µL). The cells were subsequently incubated with a 1:50 dilution of Live/Dead Blue (Invitrogen, Cat# L23105) according to the manufacturer’s instructions for 30 minutes on ice in the dark, followed by washing with 200 µL of FACS buffer (1× PBS with 5% FBS and 1 mg/mL sodium azide). Next, samples were treated with FACS buffer containing a 1:50 dilution of Human FcR block (Miltenyi Biotec, 130-059-901) and incubated for 10 minutes at room temperature (RT), followed by tetramer staining at a 1:350 dilution for 40 minutes at RT. Fluorescently labeled monoclonal antibodies were then added at the appropriate dilution determined by titration as noted below, and cells were stained for 30 minutes at 4°C. For surface staining only, cells were washed once and resuspended in FACS buffer before analysis using a Cytek Aurora Spectral Flow Cytometer. To perform spectral unmixing, reference samples were prepared using compensation beads incubated with each respective antibody for 15 minutes at 4°C. Unstained controls and viability dye reference samples utilized isolated naive CD8+ T cells. Data analysis was conducted using FlowJo version 10.10 software, with gating strategies shown in the supplemental material.

Fluorescent antibodies and compounds used:

Live/Dead Blue: Invitrogen, L34962

MART-1 Tetramer BV421: NIH tetramer facility (1/350)

SL9 Tetramer BV421: NIH tetramer facility (1/350)

SL9 Tetramer PE: NIH tetramer facility (1/350)

CD8-APC-Cy7: BioLegend, 301016 (1/250)

CD3-BUV395: BD Biosciences, 563546 (1/150)

CD62L-BV650: BioLegend, 304832 (1/200)

CD45RO-PE: BioLegend, 304206 (1/250)

CD45RA-PE-Cy7: BioLegend, 304125 (1/400)

CD95-BV510: BioLegend, 305640 (1/150)

IFNγ-BV605: BioLegend, 502536 (1/100)

TNFa-BV711: BioLegend, 502939 (1/100)

Granzyme B-Alexa Fluor700: BioLegend, 372222 (1/100)

Perforin-Alexa Fluor647: BioLegend, 353322 (1/100)

CD83-Alexa Fluor 647 (BioLegend, 305316) (1/150)

CD14-PE (BioLegend, 325606) (1/150)

### Cytokine detection by intracellular staining

A total of 5 × 10^5^ cells stimulated with Immuno-STAT or peptide-loaded mDCs were restimulated at a 1:1 ratio with T2 cells (ATCC) loaded with MART-1 26-35 (Leu27; ELAGIGILTV) or HIV-1 p17 Gag77-85 (SLYNTVATL; SL9) peptide at 10 µM with brefeldin A (BFA, BioLegend, 420601) and monensin (BioLegend, 420701) added at the manufacturer’s recommended concentration at the initiation of coculture. After 16 hours of incubation, cells were stained according to standard surface staining procedures outlined above, followed by resuspension in 2% paraformaldehyde solution (50 µL) for 10 minutes. The plate was washed with PBS (200 µL) and then resuspended in Fixation/Permeabilization buffer (50 µL) from the Foxp3/Transcription Factor Staining Buffer Set (eBioscienceTM Cat# 00-5523-00) and incubated for 30 minutes at room temperature. Next, the plate was washed three times with 200 µL of the kit’s 1× Permeabilization Buffer before resuspending in 50 µL of the intracellular staining antibody mixture in 1× Permeabilization Buffer with 5% FBS for 16 hours at 4°C. After washing, cells were resuspended in FACS buffer (200 µL) and analyzed using a Cytek Aurora Spectral Flow Cytometer. For spectral unmixing, reference samples were prepared using compensation beads incubated with each respective antibody for 15 minutes at 4°C. Unstained controls and viability dye reference samples utilized naive CD8+ T cells. Data analysis was conducted using FlowJo version 10.10 software.

### TCR immunoprofiling

Naive cells from healthy donors were expanded for 25 days with either 100 nM Immuno-STAT or peptide-loaded mDCs, following the standard *in vitro* protocol. RNA for TCR immunoprofiling was isolated from whole cultures or highly purified MART-1-specific CD8+ T cells sorted by flow cytometry from two combined replicates. For unsorted samples, 2 × 10^6^ cells were harvested from cultures stimulated with 100 nM MART-1-IST, MART-1-peptide-loaded mDCs, or left untreated, and RNA was isolated using the RNeasy Plus Micro Kit (Qiagen, Cat# 74034).

For sorted MART-1-specific CD8+ T cells, 1 × 10^7^ cells from two wells with similar tetramer percentages were collected, washed, and resuspended in FACS buffer. Cells were incubated with FcR Blocking Reagent (1:50 dilution) for 10 minutes at room temperature, followed by a 15-minute incubation with MART-1-tetramer-BV421 (1:350 dilution). After washing and filtering through a cell strainer cap, approximately 2 × 10^6^ CD8+ MART-1-tetramer-positive cells were sorted using a BD FACSAria II sorter, and RNA was immediately isolated using the RNeasy Plus Micro Kit.

All RNA samples were shipped on dry ice to Cellecta, Inc. for TCR immunoprofiling analysis using the Cellecta DriverMap Adaptive Immune Receptor (AIR) system. The quality of DriverMap AIR sequencing reads was assessed using FastQC (96). Reads were then aligned to the IMGT reference database with MiXCR (97). The preset cellecta-human-rna-xcr-umi-drivermap-air to define MiXCR alignment settings was used. The repertoire space analysis and the diversity estimation, along with data visualization, were performed using the immunarch (44) package on R. Due to high reproducibility across samples of the same treatment (i.e., untreated, IST, and mDC), we focused on the clonotypes with the greatest effect size and statistical significance. The following cutoffs were used to categorize a clonotype to be activated/inactivated when comparing two conditions: fold change ≥ 50×, false discovery rate ≤ 10^-5^, and clonotype counts (in UMI) ≥ 20.

### Epigenetic analysis

Naive cells isolated from healthy donors were expanded in 25-day cultures with either MART-1-αCD28 IST (100 nM), SL9-αCD28-IST (100 nM), or MART-1-peptide-loaded mDCs, following the standard expansion protocol outlined above. Genomic DNA was isolated using the DNeasy Blood & Tissue Kit (Qiagen, 69504) from unsorted untreated cells or MART-1-specific CD8+ T cells (DC or IST) stained with tetramer for 25 minutes in the dark at a 1:350 dilution prior to isolation by flow cytometric sorting on BD FACSAria III Cell Sorter as described above. Genomic DNA extraction and enzymatic conversion were performed using the NEBNext Enzymatic Methyl-seq Kit. Converted genomic DNA was submitted to the Hartwell Center at SJCRH for library construction and sequencing on the Illumina NovaSeq platform with 150 bp paired-end and target reads of 500 million per sample. EM Seq reads were trimmed by 10 bp on 5′ and 3′ ends as well as all Illumina adapter sequences. Trimmed reads were aligned to the hg19 genome using the BSMAP version 2.90 software. Methylation levels were called by the methratio.py script of BSMAP. Differential methylation analysis was performed by the R package DSS 2.34. Basic two-group comparisons were analyzed using a cutoff of *P* ≤ 0.01. T cell multi-potency index was calculated based on 245 CpGs and weights as published (40).

### Statistics

All statistical analysis was performed using GraphPad Prism 10.4.0. A *P* value of 0.05 was used as the cutoff for statistical significance. Significance was estimated by either one-way ANOVA followed by Tukey’s multiple comparison test or two-way ANOVA, and group differences at each ratio were computed and assessed via analyses of simple effects, using the error term and degrees of freedom from the whole design. Where indicated, significance was calculated using the Kruskal-Wallis test (*P < 0.05, **P < 0.01, ***P < 0.001, and ****P < 0.0001).

## Data Availability

Values for all data points in graphs are reported in the supplemental material. All TCR immunoprofiling and epigenetic data have been uploaded to GEO (GSE286286 and GSE286056, respectively).

## EDITOR'S NOTE

**Ed. Note:** A potential conflict of interest was identified after acceptance of this paper, and the editor in chief performed an additional final review of the paper.

## SUPPLEMENTAL MATERIAL

Supplemental figures jvi.00188-25-s0001.docx Graphical abstract and Fig. S1 to S6.

ASM does not own the copyrights to Supplemental Material that may be linked to, or accessed through, an article. The authors have granted ASM a non-exclusive, world-wide license to publish the Supplemental Material files. Please contact the corresponding author directly for reuse.
