# Supplementary material for: T-cell receptor/CD28-targeted immunotherapeutics selectively drive naive T-cell expansion to generate functional HIV-specific responses
Source: J Virol. 2025 Aug 5;99(9):e00188-25. doi: 10.1128/jvi.00188-25 (PMC12456003; doi:10.1128/jvi.00188-25)
Supplement: Supplemental figures — Graphical abstract and Fig. S1 to S6. [file jvi.00188-25-s0001.docx]

**Graphical Abstract**

**
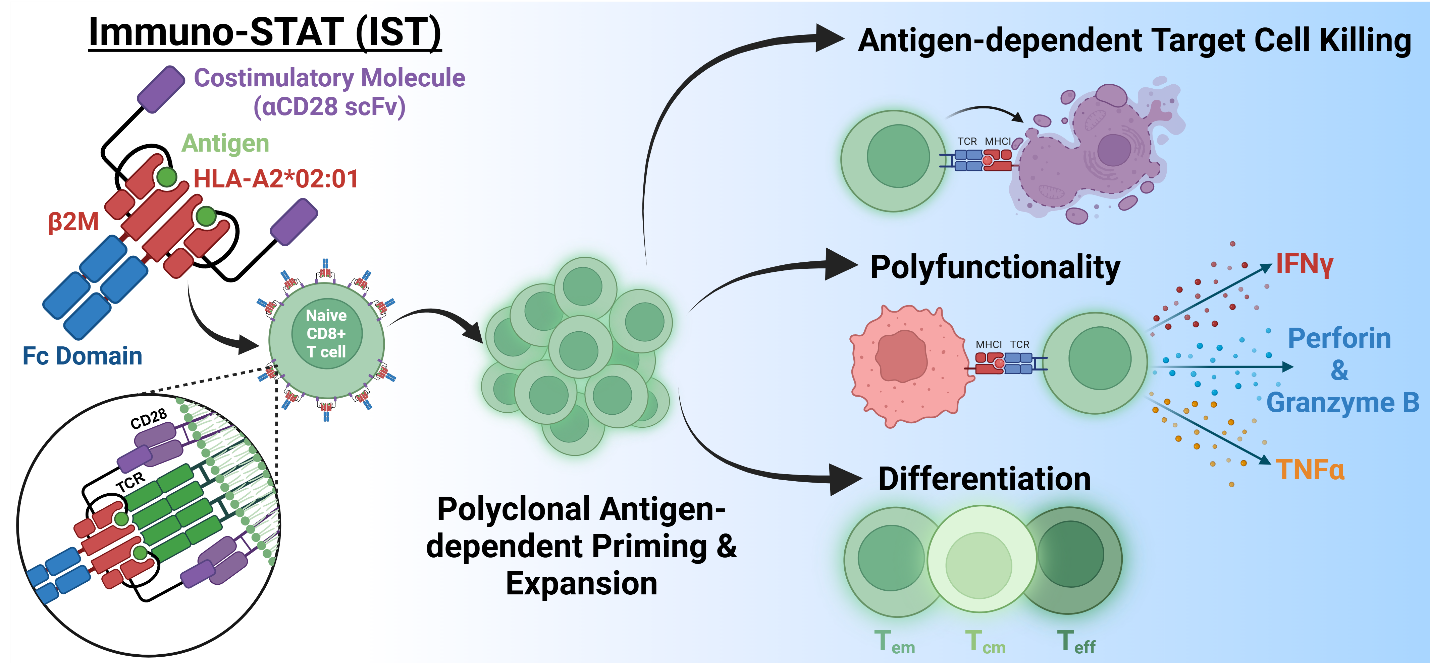
**

**Supplementary Figures**

**
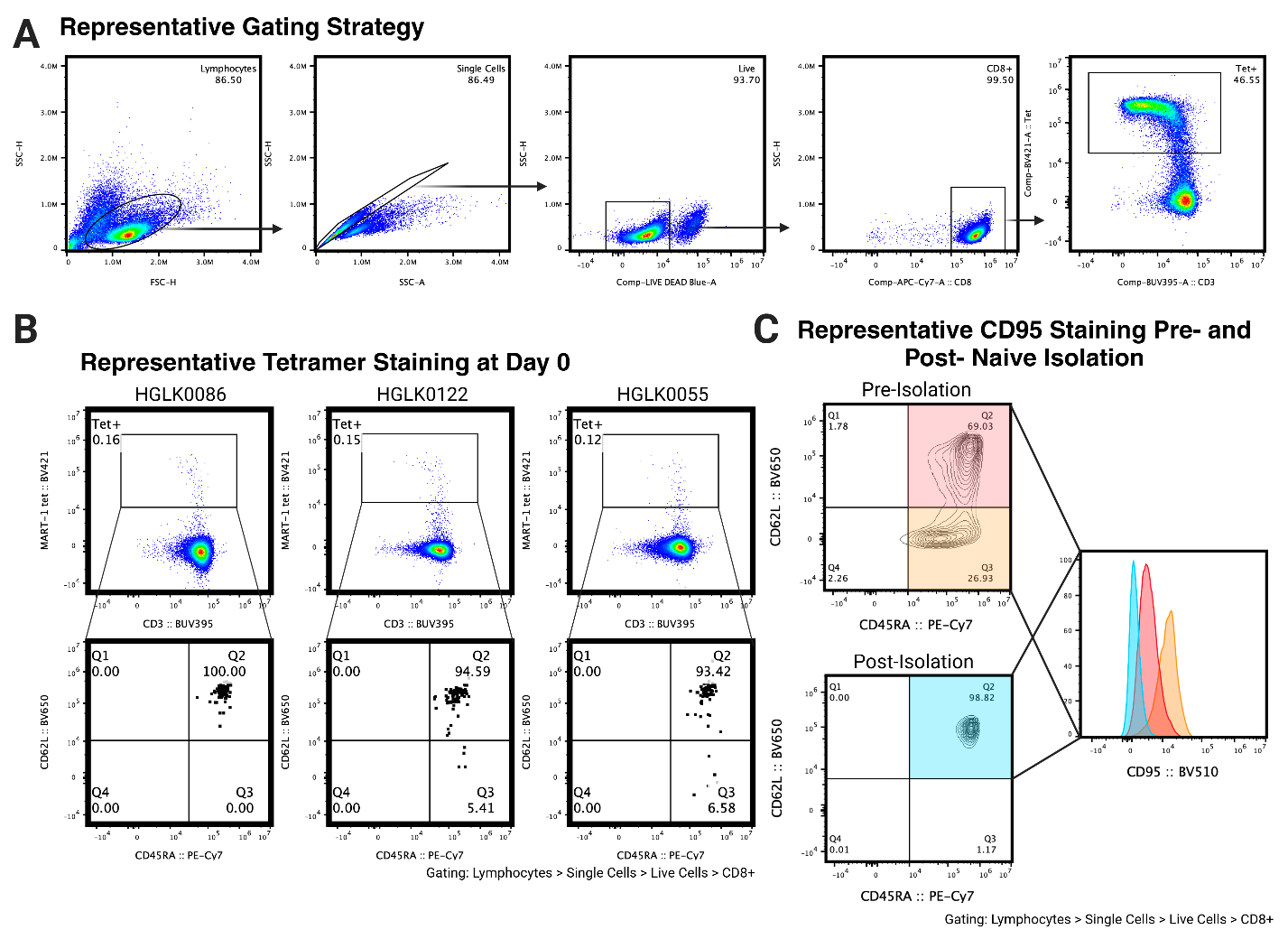
**

**Figure. S1: Gating strategy and baseline tetramer staining of MART-1**

(A) Representative tetramer+ gating strategy. For all tetramer+ quantification experiments, gating was performed as follows: lymphocytes > single cells > live cells > CD8+ cells. (B) Tetramer staining for MART-1 for each donor at day 0 as determined by flow cytometry. Tetramer positive cells (top) were gated as shown in panel A, and memory phenotype of tetramer positive cells (bottom) was determined at day 0 after naïve CD8+ T cell immunomagnetic sorting by staining CD62L and CD45RA. (C) Representative staining of CD95 before and after naïve CD8+ T cell isolation. CD8+ T cells were analyzed for memory phenotype and CD95 expression before and after naïve CD8+ T cell isolation.

**
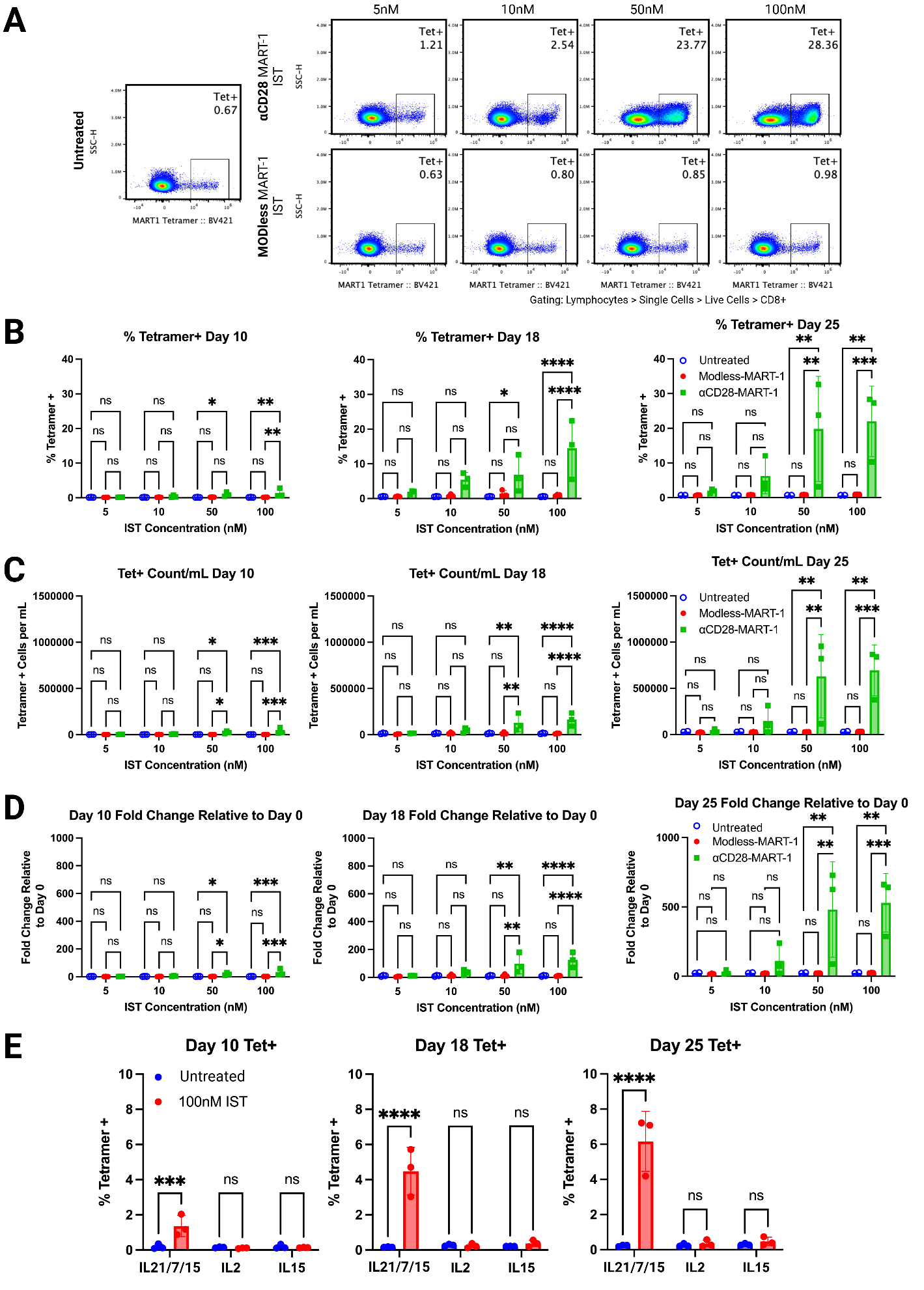
Figure. S2: Naïve CD8+ expansion by MART-1-IST is dose-dependent and requires IL21, IL7, and IL15**

(A) Representative dot plots of tetramer expansion after stimulation with IST on day 25. Isolated naïve cells were either untreated or treated with a single dose of a range of either αCD28-MART-1-IST or MODless-MART-1-IST and cultured for 25 days. (B) % Tetramer expansion at each time point. The percentage of tetramer positive cells was determined on day 10, 18, and 25 by flow cytometry. Each data point represents a biological replicate in n=1 donor from one experimental replicate. (C) Cell count at each time point. Tetramer positive cells per mL calculated by (number of tetramer positive events) / (volume of sample processed by flow cytometry in µL) * 1000. Each data point represents a biological replicate in n=1 donor. (D) Fold change at each time point. Fold change relative to day 0 is calculated by (total number of tetramer positive events per µL at day 25) / (total number of tetramer positive events per µL at day 0). Each data point represents a biological replicate in n=1 donor from one experimental replicate. (E) Expansion in three alternative cytokine conditions. Bar graphs show tetramer expansion by % total CD8+ T cells in samples treated with 100nM αCD28-MART-1-IST or untreated after day 10, 18, and 25 in either IL21 for 3 days then IL-7/15, IL-2 at 100U for the whole culture period, or IL-15 at 10ng/µl for the whole culture period. Each data point represents a biological replicate in n=1 donor from one experimental replicate. Expansion was determined via flow cytometry at the indicated time points, and significance was determined by one-way ANOVA followed by Tukey’s multiple comparisons test. Significance for (B), (C), (D), and (E) were estimated by a two-way ANOVA and group differences at each concentration were computed and assessed via analyses of simple effects, using the error term and degrees of freedom from the whole design. All statistical analysis was done in GraphPad Prism 10.4.0. (* = p<0.05, ** = p<0.01, *** = p<0.001, **** = p<0.0001).

**
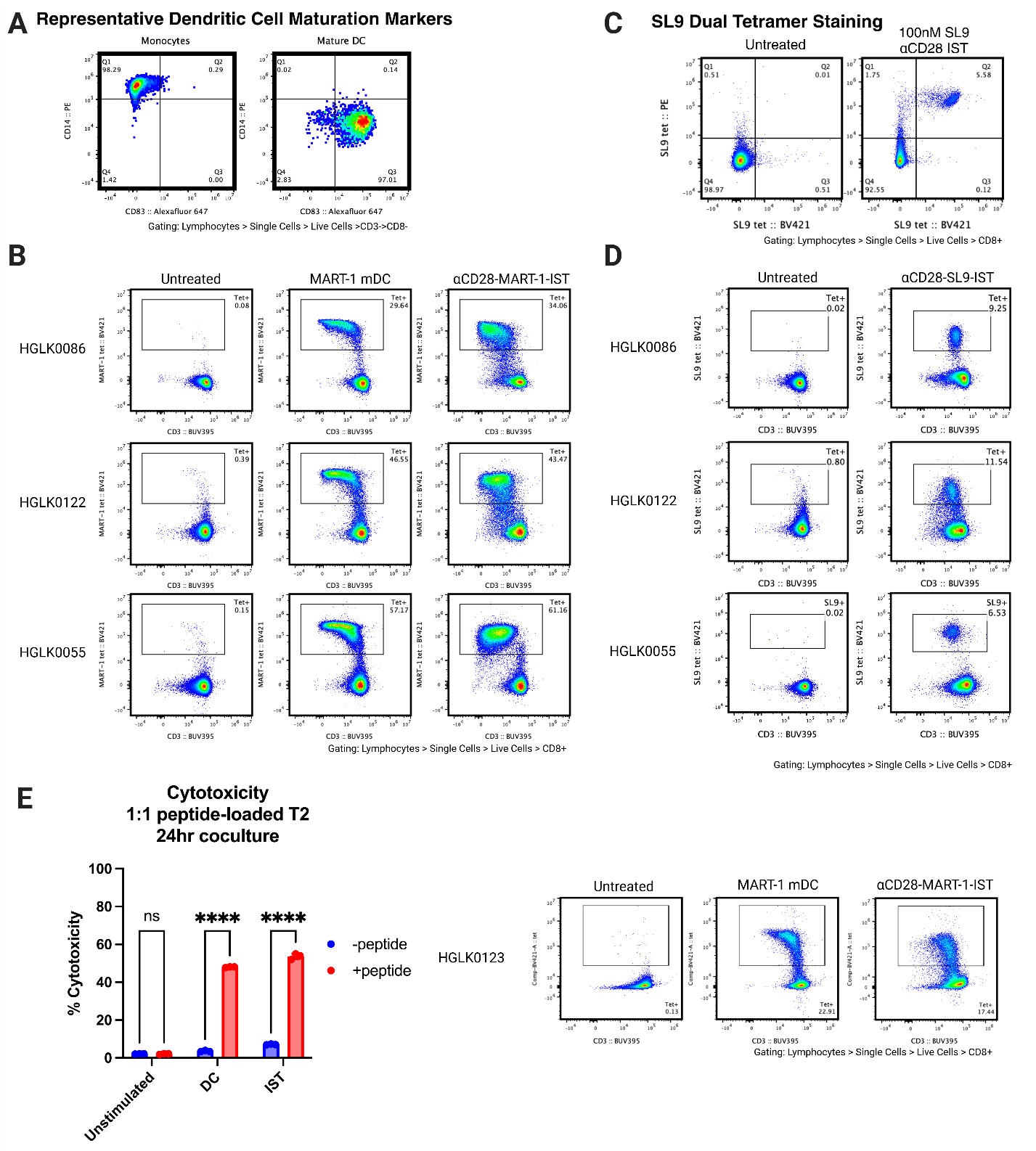
**

**Figure. S3: Dendritic cell maturation, tetramer quantification prior to functional assays, and cytotoxicity against T2 cells using flow cytometry**

(A) Representative flow cytometry plots showing maturation of dendritic cells. Monocytes (left) or mature dendritic cells (right) were stained for CD14 and CD83 to ensure mature phenotype before coculturing with naïve CD8+ T cells. (B) MART-1 tetramer expression quantified prior to functional analyses. In each donor (n=3), MART-1 tetramer positive percentage was quantified for untreated, MART-1 mDC stimulated, and αCD28-MART-1-IST samples used for cytotoxicity and immunoprofiling assays. (C) Dual staining after SL9-IST treatment. A sample treated with 100nM SL9-antiCD28-IST for 25 days was stained by both BV421 and PE conjugated SL9 tetramers. (D) SL9 tetramer expression quantified prior to functional analyses. In each donor (n=3), SL9-tetramer positive percentage was quantified in untreated and αCD28-SL9-IST-treated samples used for cytotoxicity assays. (E) 24hr flow cytometry-based cytotoxicity assay in donor HGLK0123 using DC vs IST-treated cell against T2 target cells loaded with peptide or with vehicle control. Each data point represents a biological replicate (n=3) in one donor. Flow cytometry plots from the sample used in the cytotoxicity assay is shown on the right. Significance was estimated using GraphPad Prism 10.4.0 by a two-way ANOVA and group differences at each ratio were computed and assessed via analyses of simple effects, using the error term and degrees of freedom from the whole design. (* = p<0.05, ** = p<0.01, *** = p<0.001, **** = p<0.0001)

**
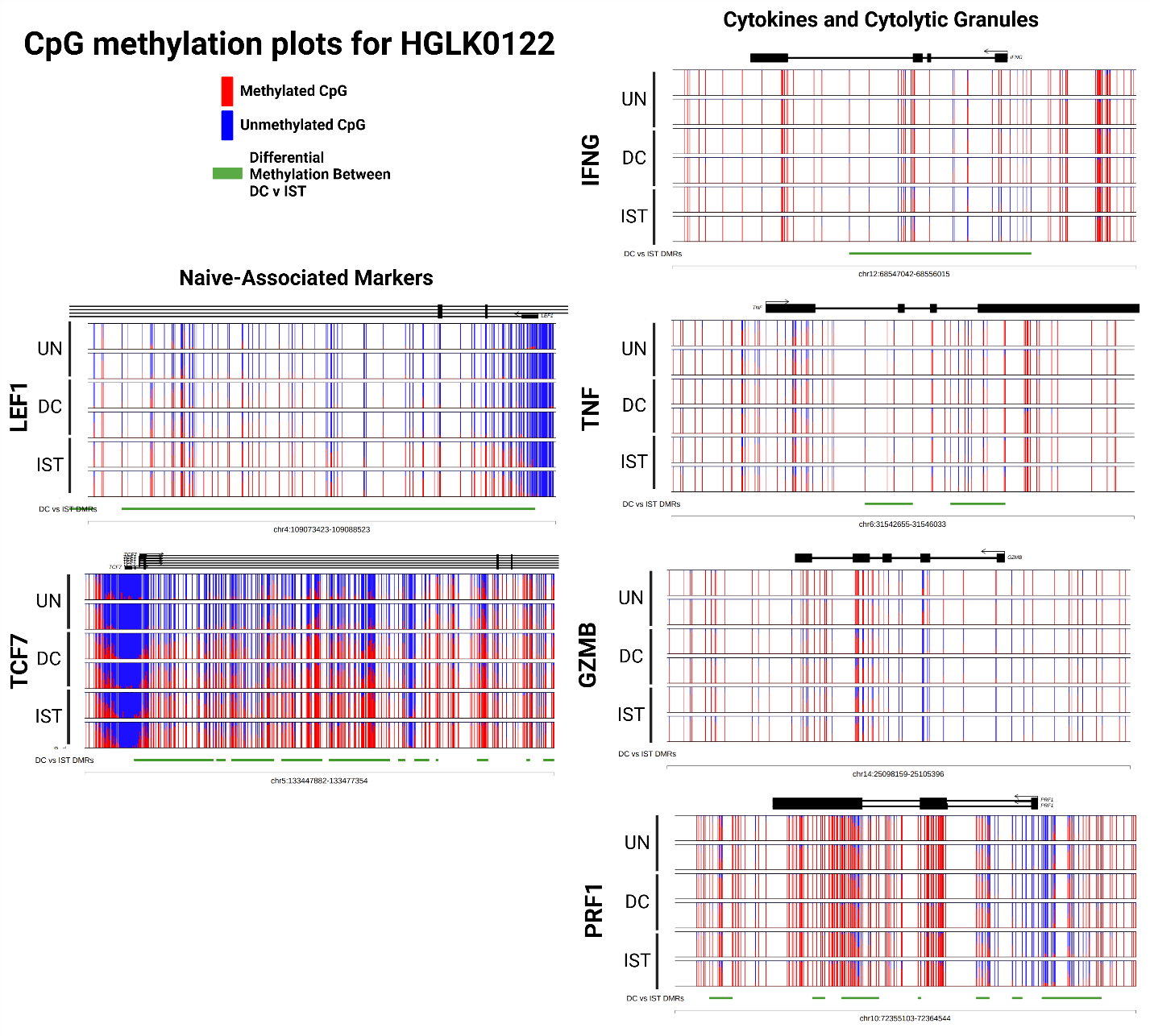
**

**Figure. S4: Epigenetic signatures of MART-1-IST treatment are consistent with differentiated cells in donor HGLK0122**

Normalized plots of CpG methylation at sites surrounding and within DMRs of effector molecules (IFNG, TNF, GZMB, and PRF1) and naïve associated transcription factors (LEF1 and TCF7) obtained from EM Seq analysis. Red and blue lines depict methylated and unmethylated CpG sites, respectively, while green lines depict significant differentially methylated regions between IST and mDC.

**
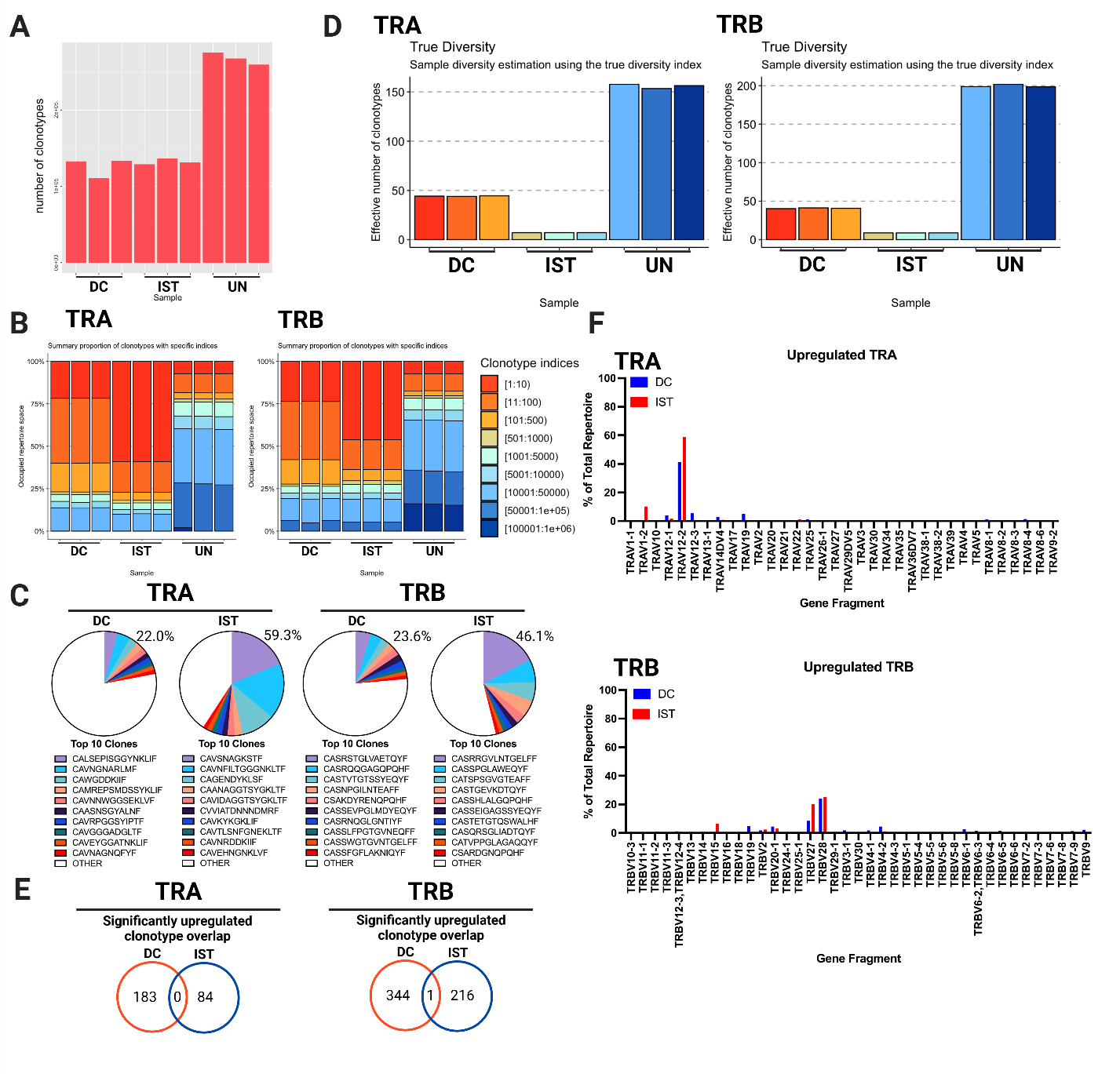
**

**Figure. S5: Treatment of naïve CD8+ T cells by MART-1-IST yields a focused TCR repertoire in bulk culture**

(A) Bar plot showing the number of unique clonotypes detected in different samples. Each bar represents a sample, with the height indicating the total number of clonotypes present. (B) Stacked bar charts representing the summary proportion of clonotypes with specific indices for TRA and TRB. Each color denotes a different clonotype index range, highlighting the distribution and diversity of clonotypes in each sample. (C) Pie charts showing repertoire space taken by top 10 identified clonotypes for TRA and TRB for each donor. The top 10 clones based on CDR3 sequencing for both TRA and TRB for both donors are plotted, showing occupied repertoire space for each individual clone and total repertoire space taken by the top 10 clones. (D) True diversity index estimation of clonotype diversity for TRA and TRB across samples. The height of each bar represents the effective number of clonotypes, illustrating the diversity within each sample. (E) Venn Diagram of overlapping significantly upregulated clonotypes based on CDR3 sequencing in DC vs IST samples. Venn diagrams were generated based on the number of significantly upregulated clonotypes compared to untreated in both DC and IST stimulated samples. CDR3 regions that appeared as upregulated in both samples take up the middle section. (F) Alpha and beta TCR gene usage of statistically upregulated clones for each sample. Each plot shows the frequency of specific gene segments, comparing the gene usage between IST and DC stimulation.

**
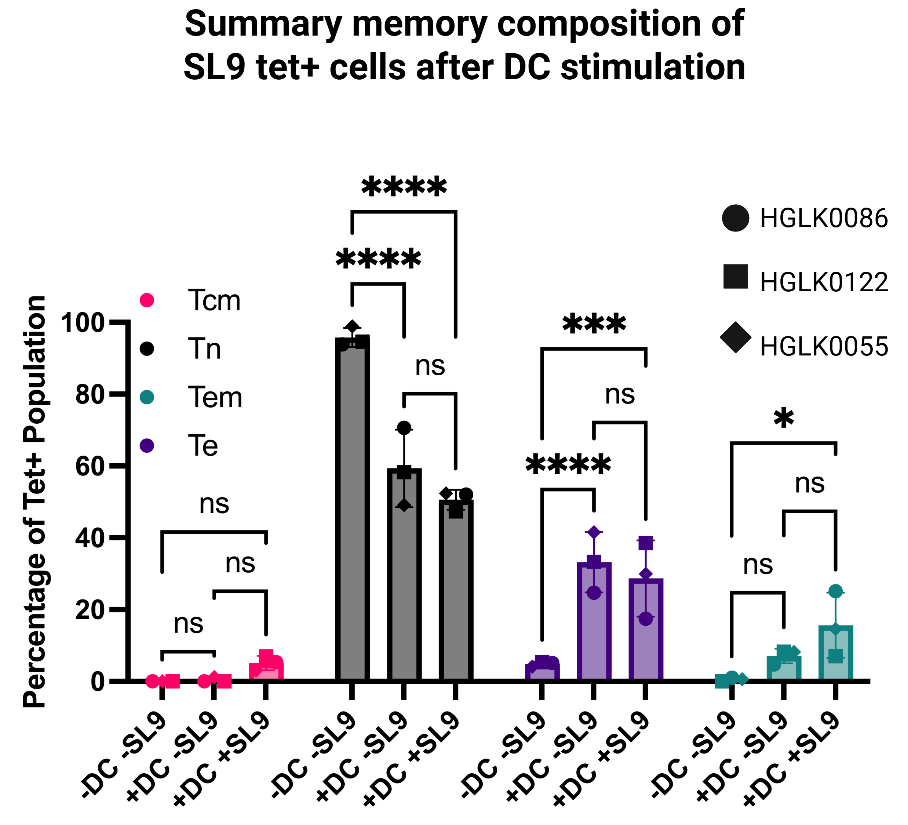
**

**Figure. S6: SL9-specific CD8+ T cells show minimal additional differentiation with SL9-loaded DCs but are significantly more differentiated than cells cultured without DCs.**

(A) Bar plot showing SL9 tetramer positive memory subtype composition for samples cultured with either no mDCs, mDCs without peptide, or mDCs loaded with SL9 peptide. Each data point represents summary data from of 3-6 biological replicates from a single donor, for a total of n=3 donors. Significance was estimated using GraphPad Prism 10.4.0 by a two-way ANOVA and group differences at each ratio were computed and assessed via analyses of simple effects, using the error term and degrees of freedom from the whole design. (* = p<0.05, ** = p<0.01, *** = p<0.001, **** = p<0.0001)
